# A Comparison of Deep Learning Techniques for Arterial Blood Pressure Prediction

**DOI:** 10.1007/s12559-021-09910-0

**Published:** 2021-08-27

**Authors:** Annunziata Paviglianiti, Vincenzo Randazzo, Stefano Villata, Giansalvo Cirrincione, Eros Pasero

**Affiliations:** 1 DET - Department of Electronics and Telecommunications, Politecnico Di Torino, Turin, Italy; 2 Lab. LTI, Université de Picardie Jules Verne, Amiens, France; 3 University of South Pacific, Suva, Fiji

**Keywords:** Arterial blood pressure, Deep learning algorithms, Electrocardiogram, Machine learning, Photoplethysmogram

## Abstract

Continuous vital signal monitoring is becoming more relevant in preventing diseases that afflict a large part of the world’s population; for this reason, healthcare equipment should be easy to wear and simple to use. Non-intrusive and non-invasive detection methods are a basic requirement for wearable medical devices, especially when these are used in sports applications or by the elderly for self-monitoring. Arterial blood pressure (ABP) is an essential physiological parameter for health monitoring. Most blood pressure measurement devices determine the systolic and diastolic arterial blood pressure through the inflation and the deflation of a cuff. This technique is uncomfortable for the user and may result in anxiety, and consequently affect the blood pressure and its measurement. The purpose of this paper is the continuous measurement of the ABP through a cuffless, non-intrusive approach. The approach of this paper is based on deep learning techniques where several neural networks are used to infer ABP, starting from photoplethysmogram (PPG) and electrocardiogram (ECG) signals. The ABP was predicted first by utilizing only PPG and then by using both PPG and ECG. Convolutional neural networks (ResNet and WaveNet) and recurrent neural networks (LSTM) were compared and analyzed for the regression task. Results show that the use of the ECG has resulted in improved performance for every proposed configuration. The best performing configuration was obtained with a ResNet followed by three LSTM layers: this led to a mean absolute error (MAE) of 4.118 mmHg on and 2.228 mmHg on systolic and diastolic blood pressures, respectively. The results comply with the American National Standards of the Association for the Advancement of Medical Instrumentation. ECG, PPG, and ABP measurements were extracted from the MIMIC database, which contains clinical signal data reflecting real measurements. The results were validated on a custom dataset created at Neuronica Lab, Politecnico di Torino.

## Introduction

Recent studies have highlighted the clinical relevance of continuous blood pressure (BP) monitoring [1]. Arterial blood pressure (ABP) is an indicator of hypertension, which is one of the most important risk factors of cardiovascular disease (CVD). For this reason, its variability is an important indicator associated with risky cardiovascular events.

Two clinical gold standards exist to measure arterial blood pressure: the invasive catheter system and the cuff-based sphygmomanometer [2]. The invasive catheter system is performed through a catheter inserted into an artery: it is used in intensive care units (ICU) to directly monitor blood pressure in the most accurate way possible and obtain samples for arterial blood gas analysis. However, only physicians and specialized nurses can perform the insertion; it is often painful and is performed by using an anaesthetic to make it more tolerable and avoid vasospasm [3]. On the other hand, cuff-based devices are the gold standard for indirect measurements and are commonly recommended by physicians. These devices offer high measurement accuracy; however, they also have several downsides: cuff size is usually too small, leading to errors in diagnosis [4] and the person using a cuff-based device has to follow a relatively strict measuring protocol to ensure that the measured values are correct. The measuring procedure can be tedious and requires time and effort; also, any physical activity (e.g. exercise) usually does not allow for simultaneous measuring of BP with a cuff [5]. Furthermore, the measuring event itself can cause white coat hypertension, commonly known as white coat syndrome: this is a condition where the patient’s blood pressure is higher when taken in a medical setting, while it is normal during daily activities. It is believed that the phenomenon is due to anxiety experienced during a clinical examination [4].

**Fig. 1:**
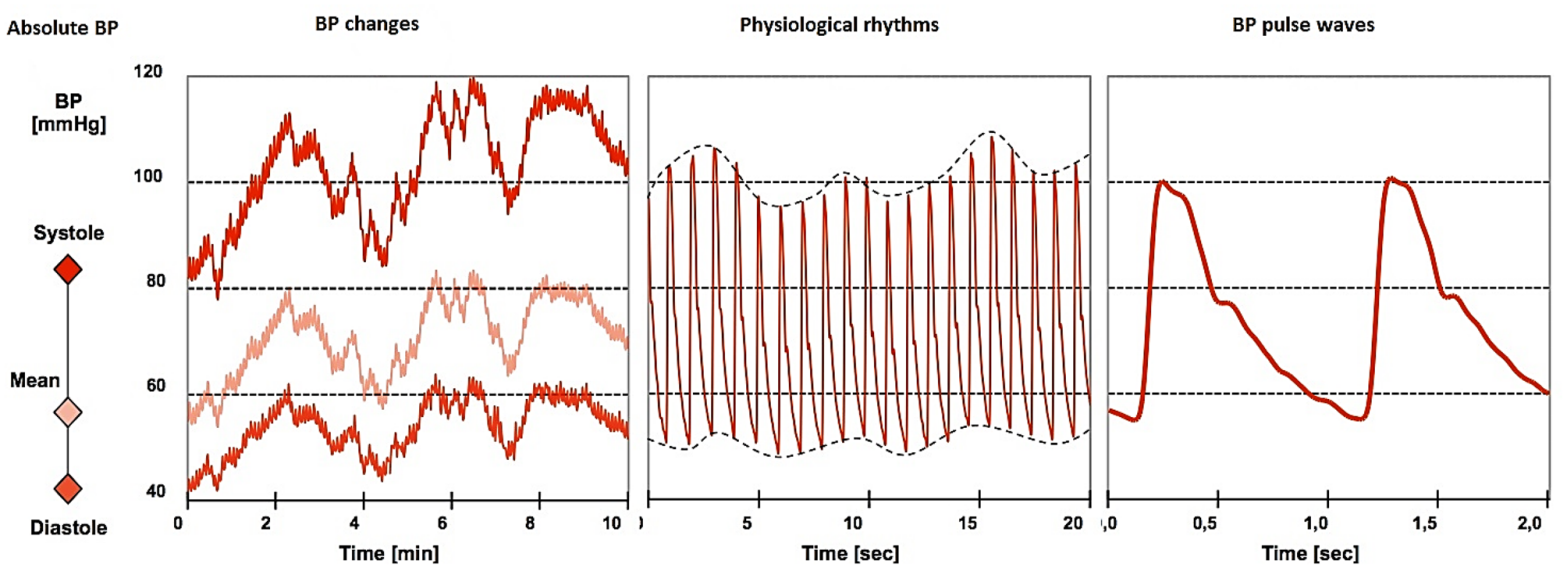
Different blood pressure information according to time resolution

Continuous non-invasive arterial pressure (CNAP) measurement combines the advantages of the two approaches. CNAP systems have different requirements for general public purposes and clinical purposes. In the public scenario, it is sufficient to measure blood pressure changes over time, while in the clinical case, the system must show not only how it varies in time, but also absolute blood pressure, physiological rhythms, and pulse waves for quality control (Fig. 1) [2].

To detect blood pressure inside an artery from the outside, several techniques have been developed. The starting point is usually volume and flow changes in the artery, which are easily collectable in the periphery (e.g. in a finger); however, these features are not linearly correlated with blood pressure, because of the non-linearity of the elastic components of the arterial wall and the non-elastic parts of the smooth muscles of the finger artery [2]. Several external techniques involve monitoring the pulse wave, which has a clear relationship with blood pressure when vessels are more relaxed or elastic, since the blood flows more slowly and with less pressure [1].

In this non-linear situation, artificial neural networks (ANNs) appear to be an ideal approach. ANNs are conceptually simple, easy to train and use, and can approximate a target function in an excellent way; however, their drawback is that the model they develop is completely obscure (black-box) and is therefore hard to analyse it [6].

The deep learning approach is gaining great popularity due to its ability to achieve state-of-the-art performance in different environments. In particular, deep neural networks have been applied to an increasing number of problems in different domains of biomedical application, such as: protein structure classification [7] and prediction [8, 9], medical image classification [10], brain computer interface systems [11], EEG classification [12], or genomic sequence analysis [13].

**Fig. 2:**
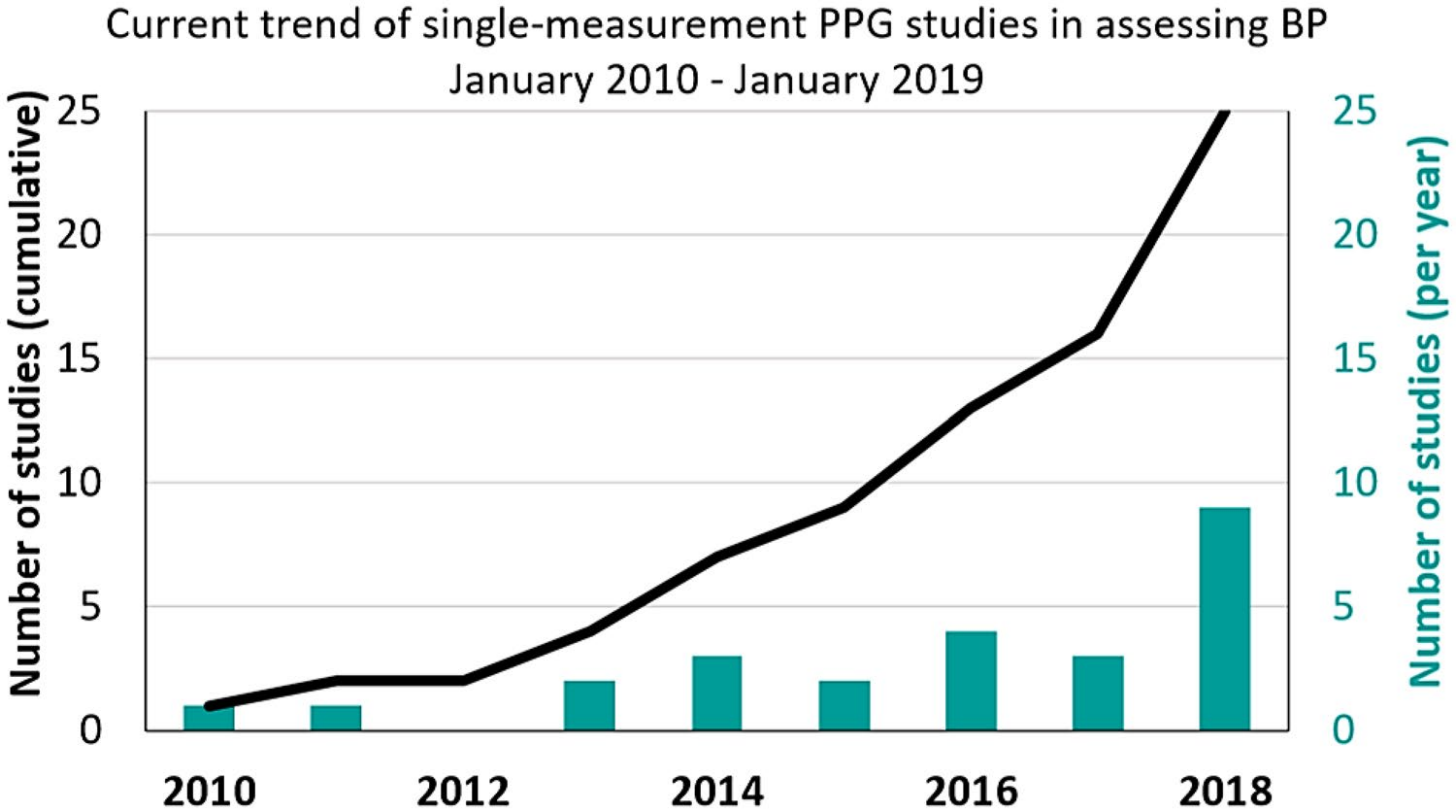
Trend of publications on PubMed database regarding single-site measurement PPG to estimate BP

As mentioned above, traditional ABP measurement techniques are either invasive or cuff-based, which are impractical, intermittent, and uncomfortable for patients. For this reason, several methods were investigated. In particular, PPG emerged as a potentially useful signal (Fig. 2) [14]; indeed, many studies point out a clear relationship between PPG and ABP. Since PPG and ECG can easily be integrated into wearable devices [15−17], they can provide the inputs of deep learning approaches for ABP estimation, as already investigated in previous works [18, 19]. Initially, indirect approaches using features derived from PPG and ECG were the most used: He et al. [20] and Shriram et al. [21] showed a strong negative correlation between ABP and pulse transit time (PTT), but pulse wave velocity (PWV) [22] and pulse arrival time (PAT) [23] were also studied. In addition, Ma et al. [22] tried to show a relationship between PWV and BP. Recently, Chua and Heneghan [23] used the mean error as the evaluation metric between the target BP value and the predicted one: the results have an error of around ± 6 and ± 4 mmHg for systolic blood pressure (SBP) and diastolic blood pressure (DBP), respectively. However, the mean error is not a suitable error metric for regression, because positive and negative differences can compensate each other in the overall mean, showing a low ME even when the individual errors are large. In Kurylyak et al. [24] it was demonstrated that ANNs can perform better than linear regression by extracting a set of features from PPG recordings obtained with the MIMIC database [25, 26]: the results have an error of around 3.80 ± 3.41 mmHg on SBP and 2.21 ± 2.09 mmHg on DBP, on a very small dataset (15,000 heartbeats were analysed, which means roughly 4 h of recordings). A complex recurrent neural network (RNN) was employed in Senturk et al. [27] on 22 features extracted from MIMIC II [28] PPG and ECG. The RMSE was 3.63 on SBP and 1.48 on DBP, i.e. a very good result.

Nowadays, thanks to the advancements in deep neural techniques, new approaches are considered which employ PPG raw signals. The idea to measure ABP using only PPG signals was already investigated in Chua and Heneghan [23], where four features were extracted from PPG signals in three different setups: rest, exercise, and recovery. This was one of the first studies based only on PPG, and it showed a good correlation between BP and some features, proving that it is possible to predict BP using only PPG based information. The results were good, but only a few healthy people were included in the cohort (i.e. with low ABP variability). Parati and Valentini [1] had a huge impact on current research: the pre-processing approach is well-suited to removing noisy signals in MIMIC, and the validation system which was adopted is the most robust applied to regression methods to estimate the ABP. However, the goal was limited to measuring the ABP starting from PPG and, therefore, ECG was not considered to obtain improved performance. This was one of the very first deep learning-based approaches: a complex neural network was developed to automatically extract and analyse both temporal and spectral features. This approach employed a huge amount of data extracted from MIMIC III and, consequently, the network was trained using a top-notch GPU cluster.

PPG-based approaches for ABP measurement are drawing ever-increasing attention in both academic and industrial fields. The approval in 2019 of the Food and Drug Administration (FDA) first cuffless device is opening up a new and interesting market.

In this study, blood pressure has been estimated by using a typical regression approach with two configurations: the first adopts the PPG signal as input, while the second employs an ECG and PPG combination as input for the neural networks whose output is the ABP.

## Methods

### Data Description and Pre-processing

**Fig. 3:**
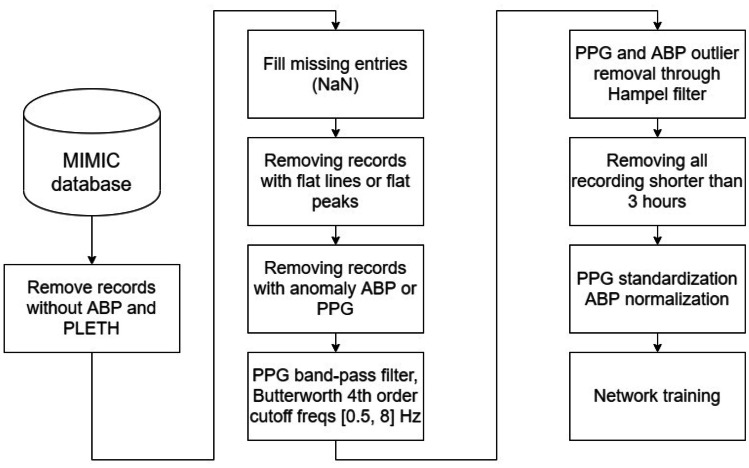
Pre-processing scheme

**Fig. 4:**
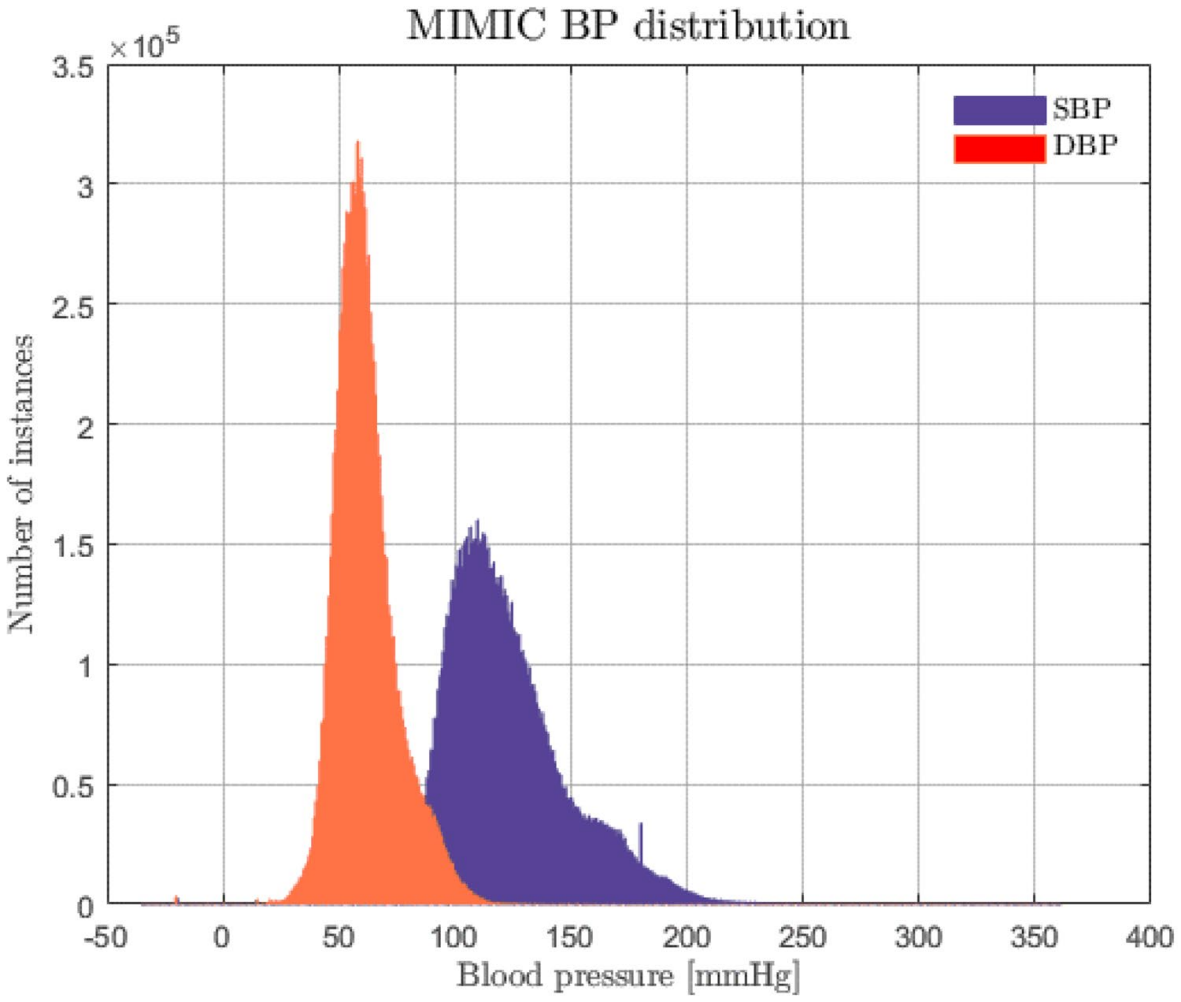
MIMIC SBP (purple) and DBP (orange) distributions

The MIMIC [25, 26] database was exploited to evaluate how PPG, ECG, and ABP are linked. It was chosen, because it is representative of a wide range of pathophysiologies that result in sudden blood pressure changes [29]. It consists of different physiological signals recorded from 121 ICU patients: data included signals and periodic measurements obtained from a bedside monitor, that is from clinical data obtained from the patient’s medical record. Acquisitions ranged from 1 to 80 h depending on patients. Data obtained from the bedside monitors were divided into 10-min segments, which can then be assembled without gaps to form a continuous recording. The ECG, PPG, and ABP signals were sampled at 125 Hz with 12-bit precision and negligible jitter [24]. MIMIC was extracted via WFDB [30], a Python library supported by Physionet [31]; subsequently, each recording without the requested signals was discarded. Figure 3 shows the pre-processing scheme, based on the histogram of SBP and DBP distributions. To extract the SBP and DBP values, the algorithm developed by Elgendi et al. [32] was applied to the raw dataset. From the graph (see Fig. 4), several problems can be identified: first of all, there are negative BP values, some too high values and an unusual peak at 180 mmHg. Finally, both distributions are heavily skewed towards physiological values, but SBP appears to have much larger support.

Since MIMIC was organized in 10-min recordings, in order to maintain consistency, the following pre-processing scheme (see Fig. 3) was applied to each 10-min segment:

- Replacement of NaN values with the closest available data;
- Deletion of records with “flat lines” in the ABP or PPG;
- Deletion of acquisitions where more than 5\% of the ABP or PPG peaks were *flat peaks*;
- Removal of records containing abnormal ABP or PPG;
- PPG filtering using a 4th order Butterworth filter;
- PPG and ABP outlier removal through Hampel filter;
- Removal of patients’ recordings with < 3 h of recording time;
- Standardization of the PPG and ABP normalization.

NaN values were simply replaced with the closest value because in some cases there were long time intervals with missing values, so it was impossible to reconstruct the missing signal. This operation did not bias the values; indeed, NaNs were associated with *flat lines*, which were managed in the subsequent step of the pre-processing scheme.

**Fig. 5:**
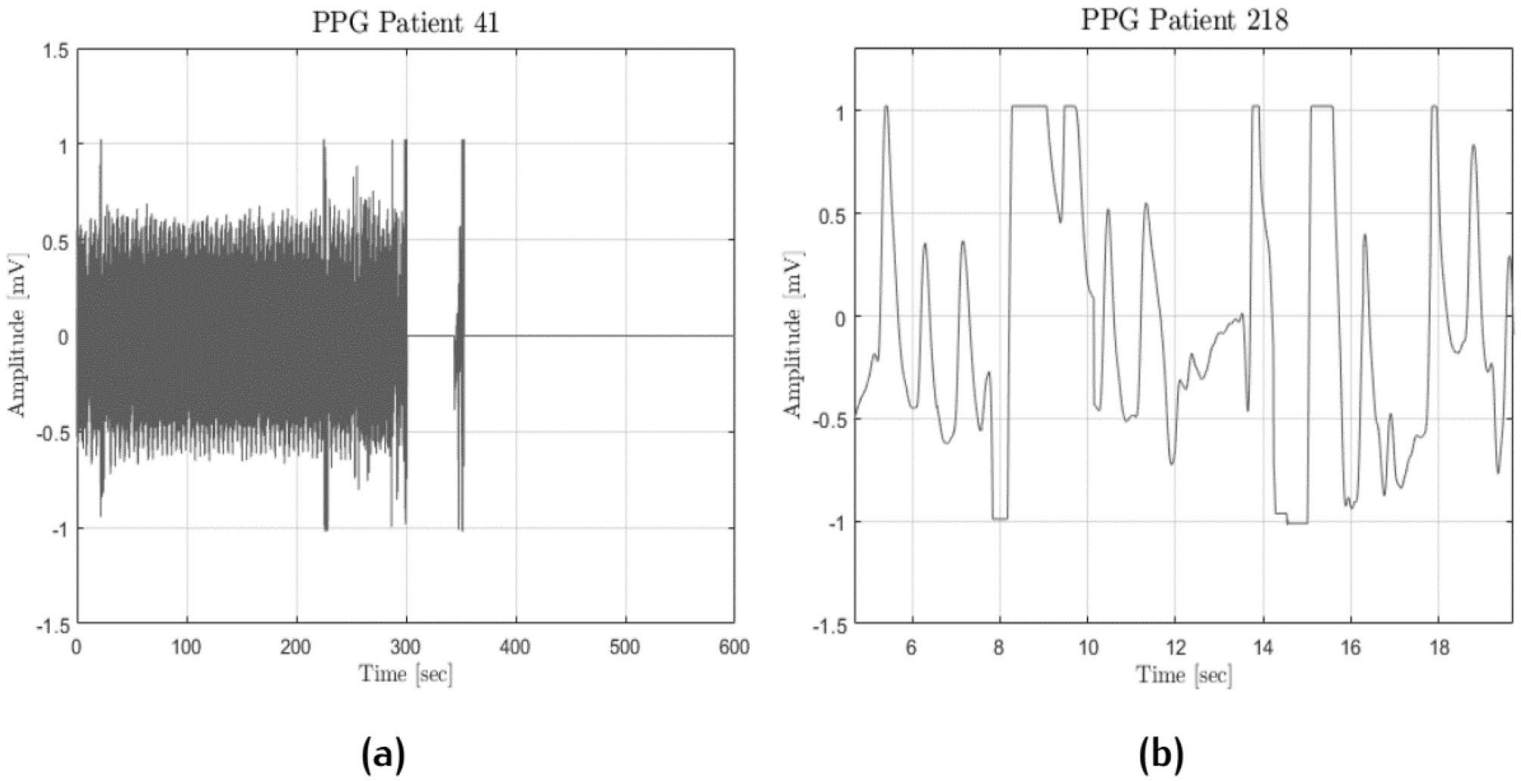
Frequent PPG anomalies: flat lines (**a**) and flat peaks (**b**)

It was necessary to exclude low-quality recordings, i.e. those containing the so-called *flat lines* and *flat peaks*, which are recording errors mostly due to sensor problems, e.g. a simple disconnection. Flat lines (see Fig. 5a) are long periods of time where the same value is always detected, while flat peaks (see Fig. 5b) are peaks with a flattened tip.

**Fig. 6:**
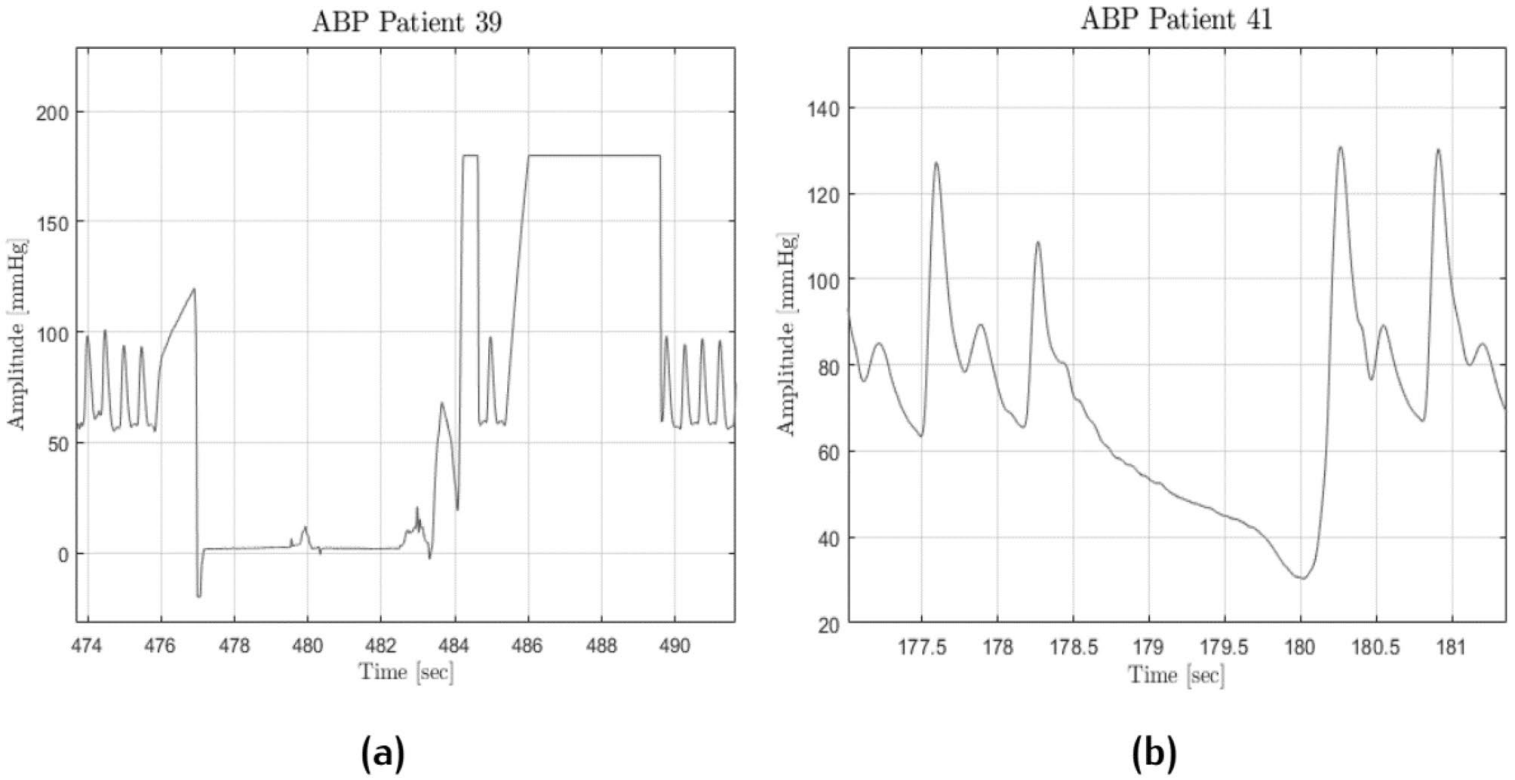
ABP anomalies: negative BP followed by flat peaks (**a**) and no heartbeat for almost 2 s (**b**)

Subsequently, anomalies in ABP signal were managed: within the 10-min recording, ABP signals should always range between a minimum of 15 and a maximum of 300 mmHg. In addition, a check on pressure and PPG signal derivatives was introduced; in particular, the pre-processing scheme deleted recordings that had the first derivative always larger than zero or always less than zero for more than 170 samples. In summary, the pre-processing scheme eliminated all recordings in which the trend was either increasing monotonously or decreasing monotonously for at least 1.36 s. This test was necessary because several patients showed either negative pressure (see Fig. 6a) or strange cardiac cycles (see Fig. 6b).

**Fig. 7:**
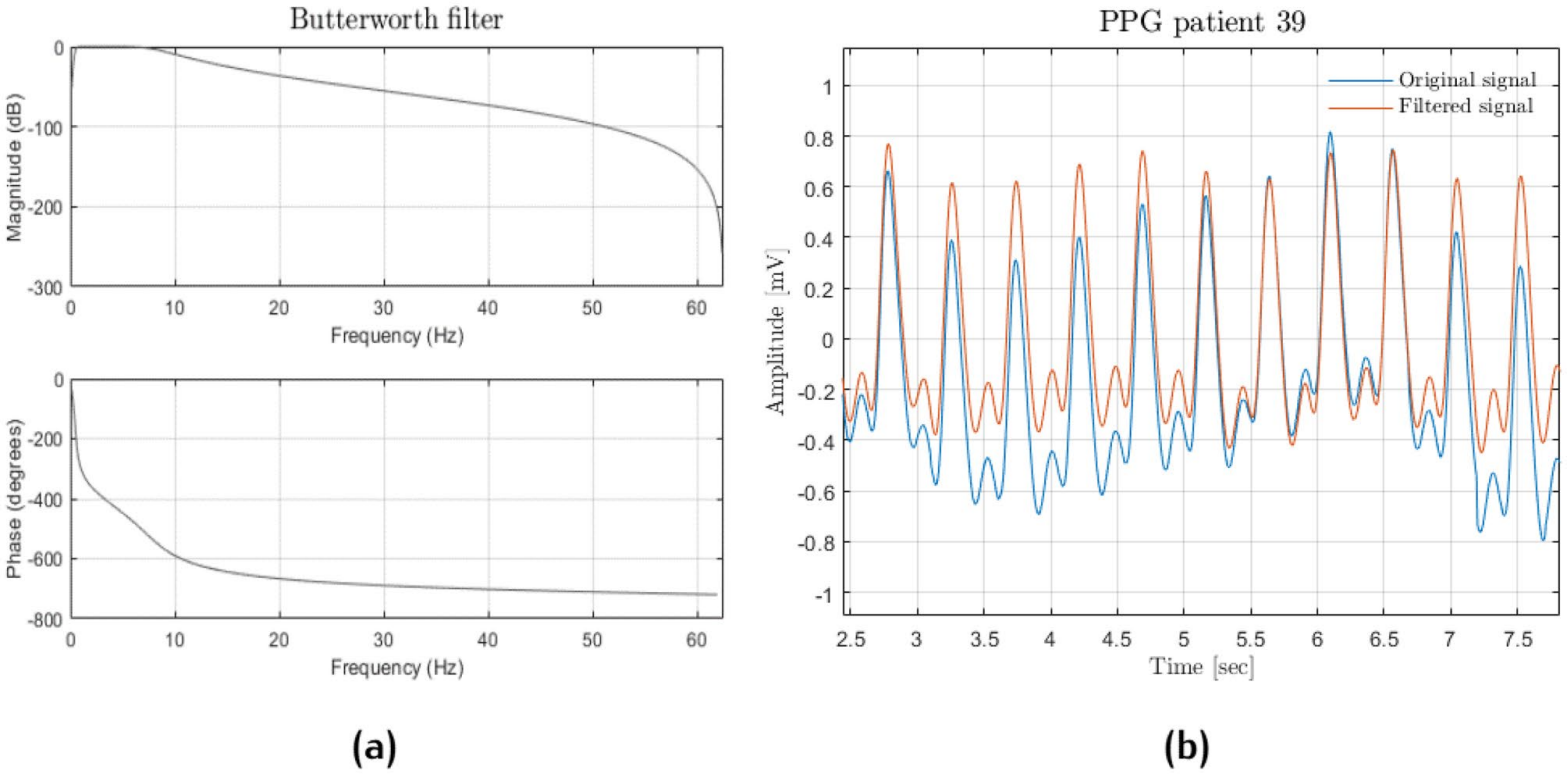
Butterworth filter: frequency response (**a**); original (light blue) and filtered (orange) signal comparison (**b**)

**Fig. 8:**
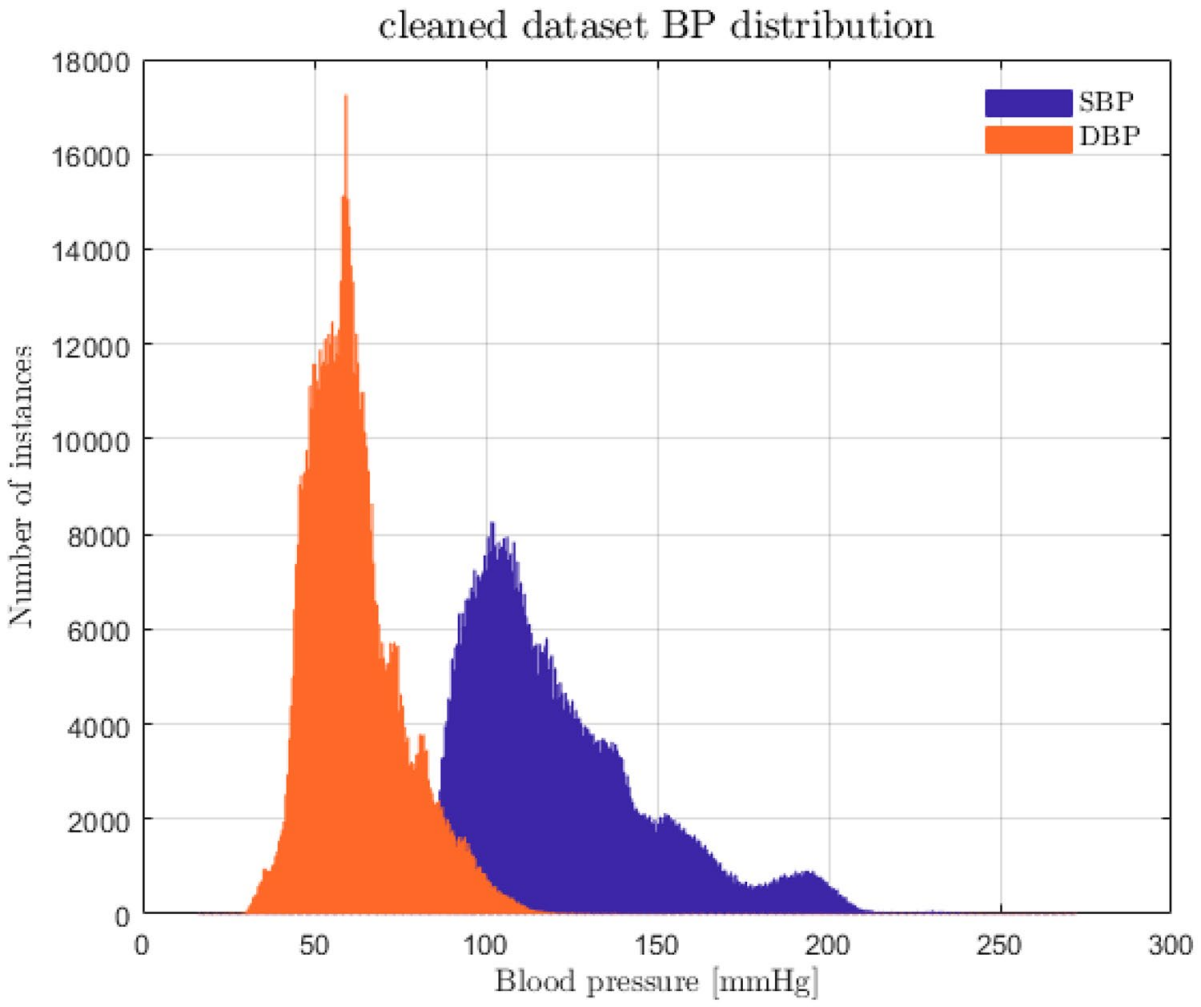
Dataset with only PPG: SBP (purple) and DBP (orange) distributions

The remaining PPG recordings were filtered through a band-pass 4th order Butterworth filter with a bandwidth between 0.5 and 8 Hz (see Fig. 7). Afterwards, both PPG and ABP signals were input to a Hampel filter. The Butterworth frequencies were chosen because anything below 0.5 Hz is due to baseline wandering, while beyond 8 Hz, the signal is made up of high-frequency noise. For computational reasons, patients with < 190 min of recordings were discarded, while only the first 190 min were considered from those patients with longer recordings. Figure 8 shows systolic and diastolic BP distributions; it is apparent that they are heavily skewed toward physiological values.

Finally, the PPG signal was standardized and the ABP signal was normalized. Since the output was normalized, predictions made by the networks had to be de-normalized to map the output into a physiological ABP range. In this sense, the statistics employed may not have been valid for all the possible test sets. This assumption needs to be verified on a real-case scenario. However, the ABP range is a very limited interval; therefore, new cases are most likely to have the same dynamic as the training set which was adopted.

Target variables often need rescaling; for this reason, a target with a large spread of values may result in large error gradient values, which cause weight values to change dramatically and the learning process to be unstable.

### Dataset with PPG and ECG

Because ABP is strictly related to ECG, several techniques for predicting ABP were developed starting from ECG and PPG (for instance, Pulse Transit Time). Thus, a second dataset was created to study whether also using ECG could be of help with deep learning approaches. To get the largest possible dataset, ECG lead V was used, because it is the most frequent ECG lead recorded in the MIMIC database.

**Fig. 9:**
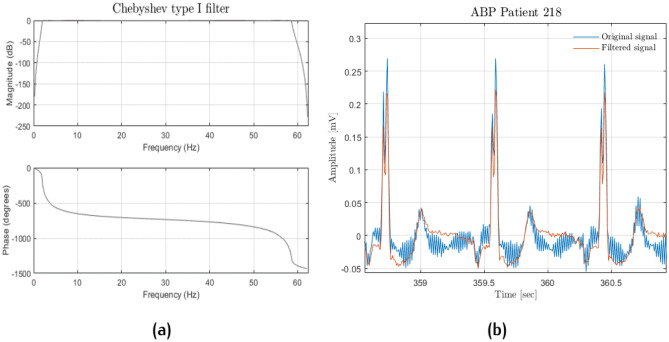
Chebyshev filter: frequency response (**a**); original (light blue) and filtered (orange) signal comparison (**b**)

**Fig. 10:**
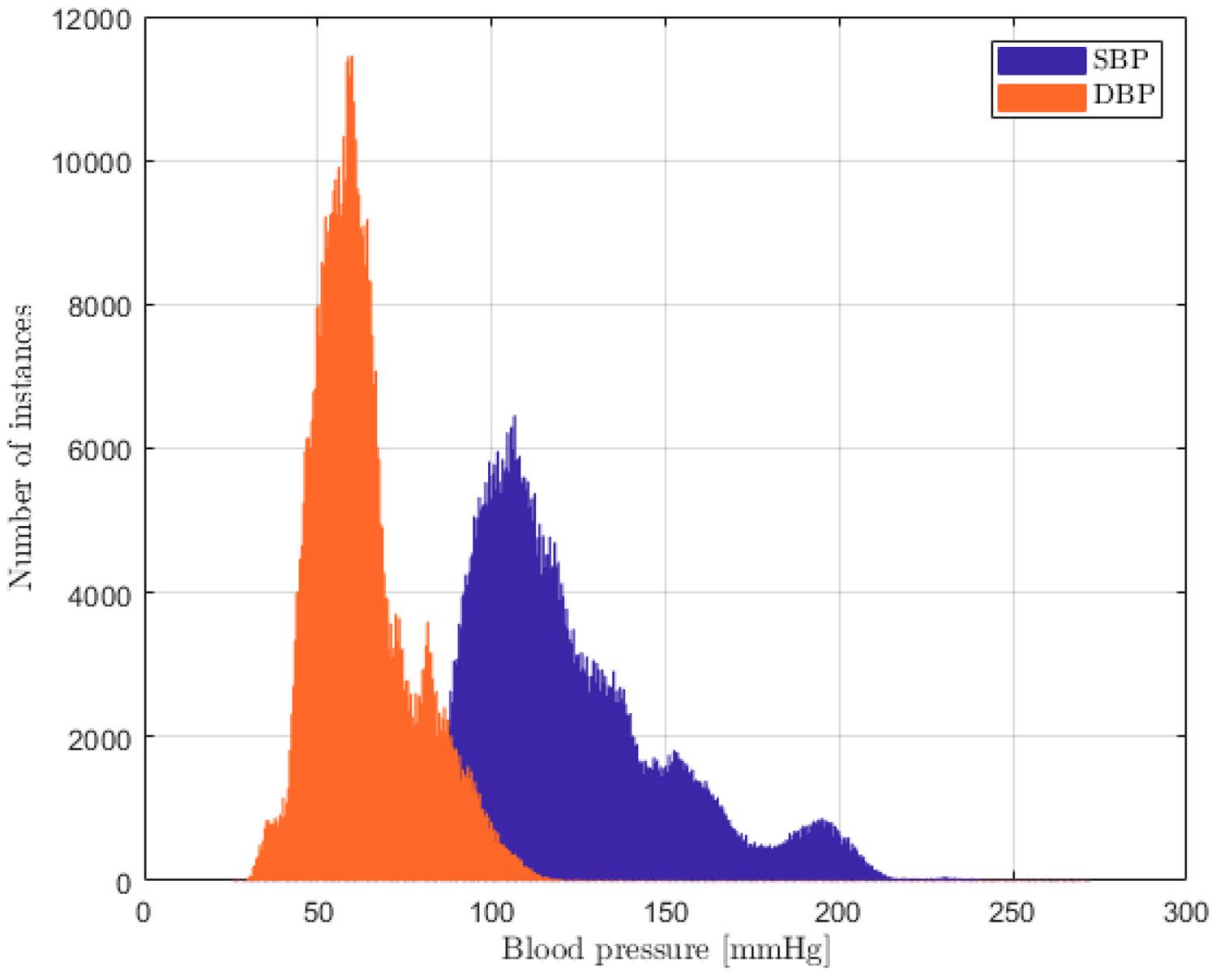
Dataset with both ECG and PPG: SBP (purple) and DBP (orange) distributions

The pre-processing scheme was the same as the one before, extended to ECG. The ECG signal was filtered with an 8th order passband Chebyshev type 1 filter with a cut-off frequency of 2 Hz and 59 Hz to avoid motion artefacts and alternating current artefacts (see Fig. 9). Out of all the MIMIC dataset patients, only 51 had at least PPG, ECG lead V, and ABP. After pre-processing, the resulting dataset was made up of 40 patients. As shown in Fig. 10, also in this dataset, SBP and DBP distributions are skewed towards physiological values.

### Metrics

Historically, the most commonly used metric for regression task is the root-mean-squared error (RMSE), which measures how large the difference is between the squares of the predicted and the target values. Since it is a squared difference, this metric gives more weight to large errors. It is also called L2 norm and corresponds to the Euclidean norm:

1 $$RMSE\left(X,h\right)= \sqrt{\frac{1}{m}\sum_{i=1}^{m}{(h({x}^{\left(i\right)})-{y}^{(i)})}^{2}}$$

RMSE is, usually, the preferred metric; however, in some contexts, some other functions can be employed. If there are many outliers, the mean absolute error (MAE) could be a more accurate performance index:

2 $$MAE\left(X,h\right)=\frac{1}{m}\sum_{i=1}^{m}|h\left({x}^{\left(i\right)}\right)-{y}^{\left(i\right)}|$$

**Fig. 11:**
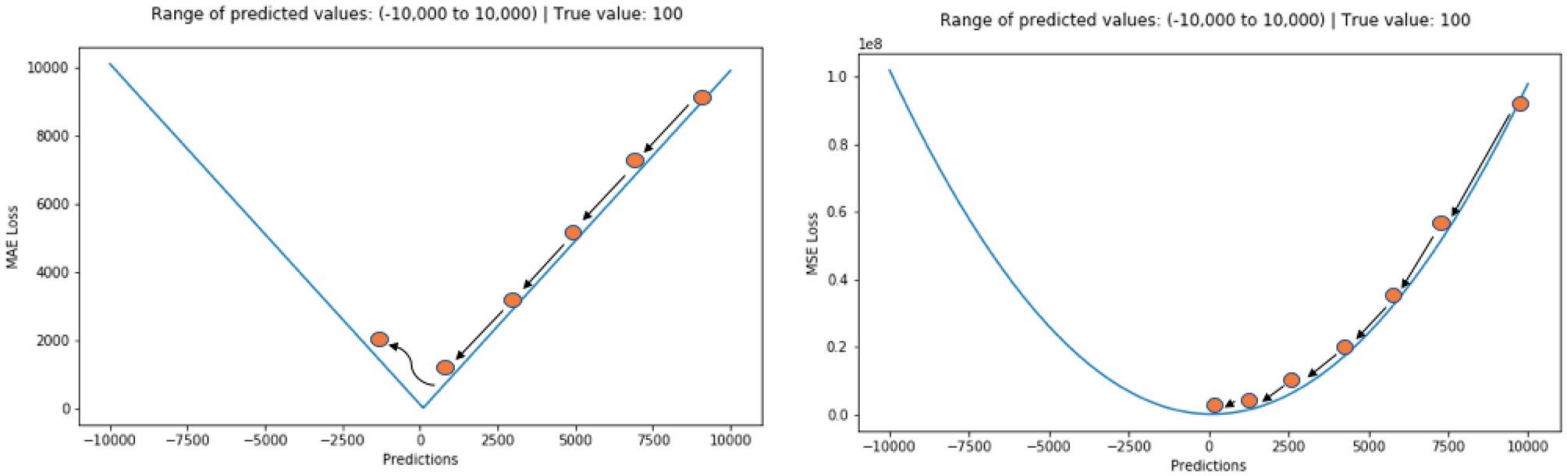
Gradient descent on MAE (left) and MSE (right)

**Fig. 12:**
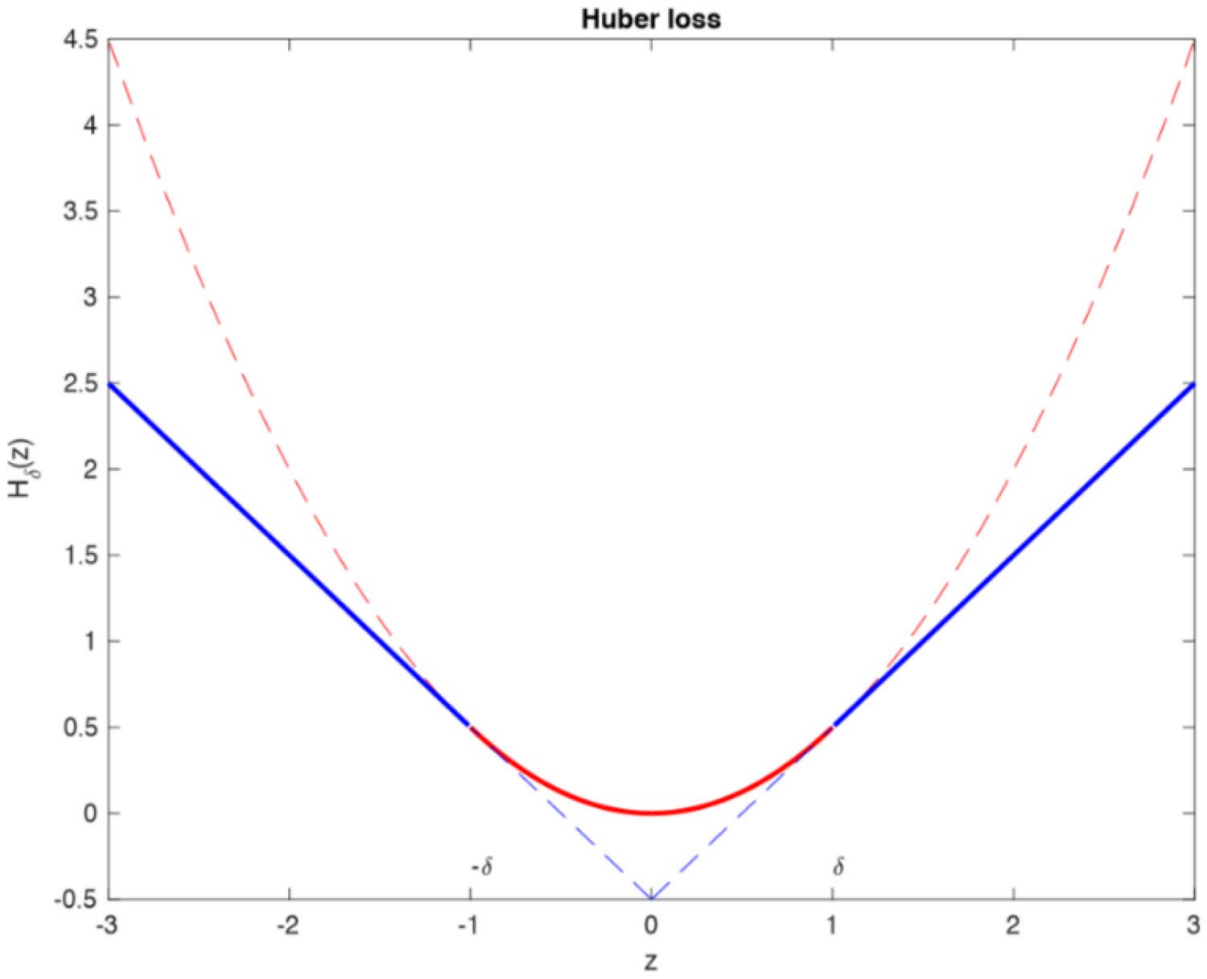
Huber loss; red dashed line is MSE, blue dashed line is MAE, δ = 1

MAE (also called Manhattan norm or L1 norm) measures the distance between two vectors, i.e. the predicted and target value ones. However, MAE cannot be utilized as loss function for a neural network (NN) because its gradient is always the same; thus, it will be large even for small loss values (see Fig. 11). For this reason, RMSE was chosen. In order to overcome problems regarding robustness, the selected loss function was the Huber loss (see Fig. 12), which is also less affected by outliers. Like the RMSE, it is differentiable in zero, but introduces another hyperparameter that needs to be tuned (δ) [33]:

3 $${L}_{\delta }(y,f(x))=\left\{\begin{array}{c}\frac{1}{2}{(y-f(x))}^{2} for \left|y-f\left(x\right)\right|\le \delta \\ \delta \left|y-f\left(x\right)\right|-\frac{1}{2}{\delta }^{2} otherwise.\end{array}\right.$$

### Tested Neural Architectures

#### ResNet

The Residual Network (say ResNet) is an architecture inspired by the pyramidal cells in the cerebral cortex, developed by Kaiming He et al., and originally used for image classification [34]. This architecture was created to overcome the difficulty in training deep neural networks with vanishing/exploding gradients and degradation of accuracy. The former problem was solved by normalizing initialization and intermediate layers. The latter issue involves saturated accuracy that degrades rapidly; because degradation is not caused by overfitting, adding more layers to a suitably deep model leads to higher training error.

**Fig. 13:**
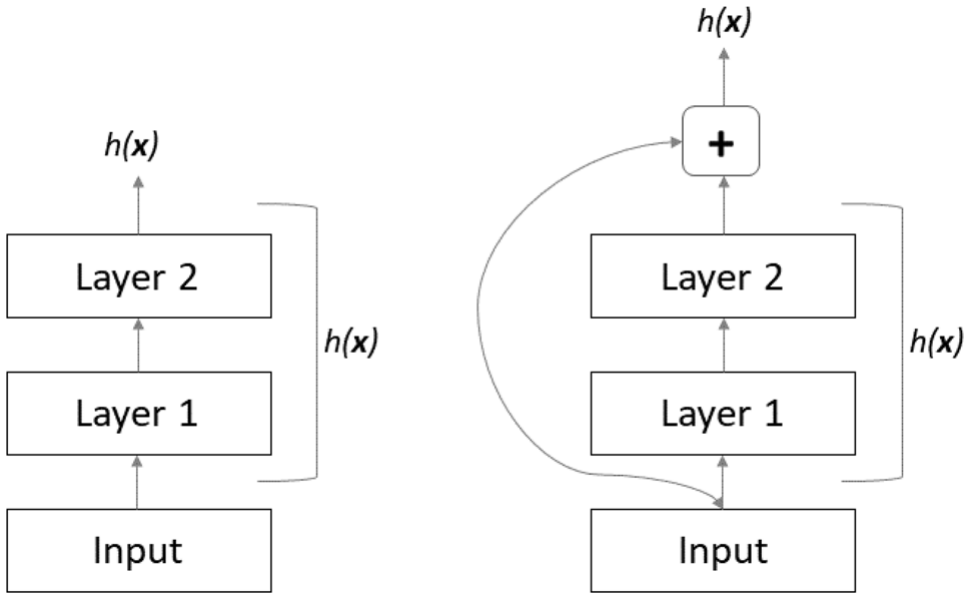
Skip connection

In order to avoid degradation, *skip connections* were introduced (see Fig. 13): the signal fed to a layer was also added to the output of the layer located a bit higher up the stack. This new technique allowed very deep networks to be trained like the original ResNet, which was a convolutional neural network (CNN) composed of 152 layers. There are many variants of this net depending on depth.

In general, a neural network is trained to make it model a target function $h(x),$ also called underlying mapping; however, when the network is deep, it is difficult to optimize it. For this reason, the input x is added to the output of the network forcing the network to learn the residual map $f\left(x\right)= h\left(x\right)-x$. Such an approach is called *residual learning*. When a regular neural network is initialized, its weights are close to zero, and therefore, the network just outputs values close to zero. However, if there is a skip connection, the resulting network just yields a copy of its inputs; in other words, it initially models the identity function. If the target function is fairly close to the identity function (as often occurs), this will speed up training time considerably [35]. Moreover, if there are many skip connections, the network can start making progress even if several layers have not started learning yet. Thanks to skip connections, the signal can easily make its way across the whole network. The deep residual network can be seen as a stack of residual units, where each residual unit is a small neural network with a skip connection [35]. Each residual unit is composed of two convolutional layers, with Batch Normalization and ReLU activation, using $3 \times 3$ kernels and preserving spatial dimensions.

#### WaveNet

WaveNet is an architecture developed in Oord et al. [36] and was originally designed to operate directly on raw audio waveforms. In its simplest variant, it is just a stack of convolutional layers without pooling layers and with a particular type of padding called *causal*, which allows the output to have the same time dimensionality as the input. Since this model does not require recurrent connections, it is typically faster to be trained than RNN, especially when applied to very long sequences. However, one of the problems of causal convolutions is that they require many layers, or large filters to increase the receptive field.

**Fig. 14:**
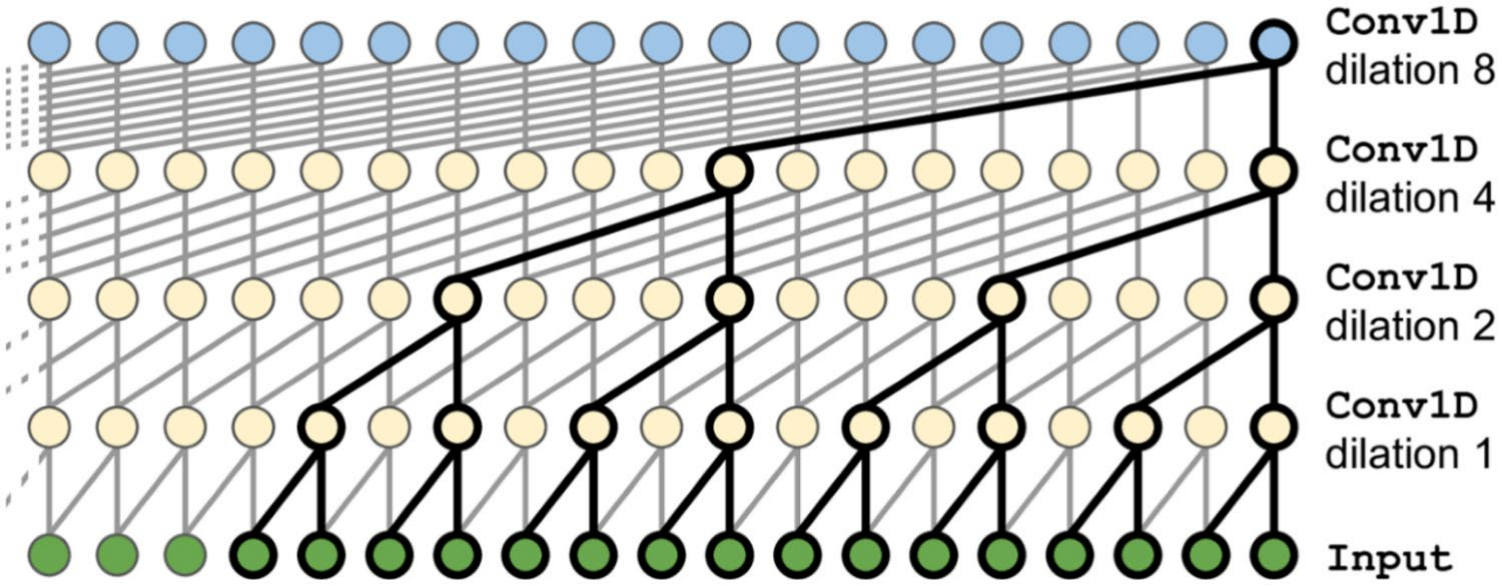
Dilated convolution

In order to solve this problem, the WaveNet utilizes a dilation rate (see Fig. 14), which represents how spread apart each neuron inputs are. A dilated convolution is a convolution where the filter is applied over an area larger than its length by skipping input values with a certain step. In this way, the lower layers learn short-term patterns, while the higher layers map long-term ones. Thanks to the doubling dilation rate, the network can process extremely large sequences very efficiently [35].

#### LSTM

**Fig. 15:**
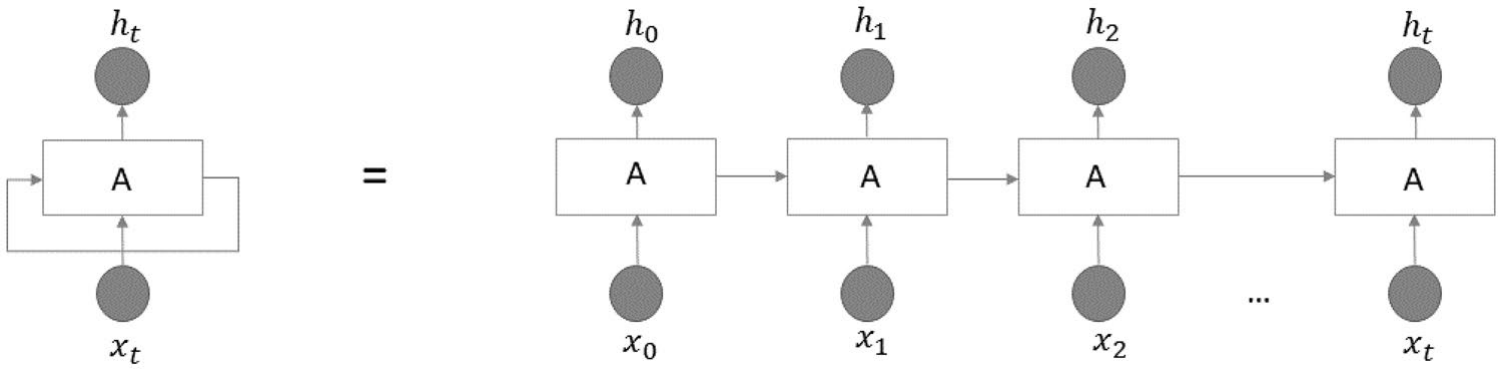
RNN unrolled through time

Unlike humans, traditional neural networks restart thinking from scratch every second; i.e. they do not have memory. This is crucial in certain tasks, like reading, where the meaning of each word is based on the previous ones. For this reason, recurrent neural networks (RNNs) employ loops (see Fig. 15) to make information persistent over time.

As shown in Fig. 15, at each time step, a RNN block receives inputs, produces an output, and then it sends the output back to itself. The network will use the last output together with the next input to produce a new output. An RNN can be thought as multiple copies of the same network, each passing a message to a successor. Since the output of a recurrent neuron at time step is a function of all the inputs from previous time steps, it has a form of memory; indeed, the part of a neural network that preserves some state across time steps is called a *memory cell*. RNNs are trained using the backpropagation through time; however, when the input sequence is long the unrolled network becomes deep. As a consequence, like every deep NN, it suffers from unstable gradients; moreover, it may forget the first input of the sequence. For these reasons, several types of memory cells were studied.

**Fig. 16:**
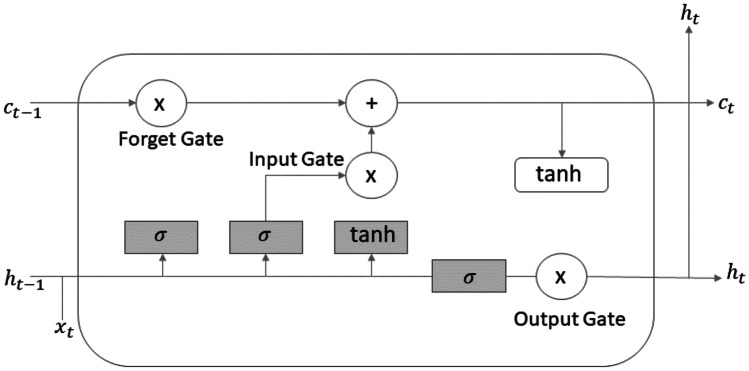
LSTM cell

Figure 16 shows the most famous RNN, the Long Short-Term Memory (LSTM) cell, which is explicitly designed to avoid the long-term dependency problem. These kinds of cells have a long-term state, where, at every iteration, the network learns what to store and what to read from it. The working memory is called the hidden state ${h}_{t}$.

The cell regulates its state using the gates: at first, there is a *forget gate* where some memories are dropped, then the memories are replaced with new ones selected by the input gate. The forget gate is rule by the following:

4 $${f}_{t}=\sigma \left({W}_{f}\left[{h}_{t-1},{x}_{t}\right]+{b}_{t}\right)$$

where $W$ presents the weights vector, $b$ represents the bias, σ represents the sigmoid function, and $t$ represents the current instant.

One copy of the new state is sent to the next iteration; the other one is passed through a *tanh* function and filtered by the *output gate*, as follows:

5 $${o}_{t}=\sigma \left({W}_{o}\left[{h}_{t-1},{x}_{t}\right]+{b}_{o}\right)$$

This is combined with the current inputs and the previous outputs to create the new output. The hidden state contains information on previous inputs, and it is also used for predictions. Its output is given by

6 $${h}_{t}={o}_{t}*\mathrm{tanh}\left({c}_{t}\right)$$

The *input gate* recognizes important inputs and stores them into the long-term state, the forget gate deletes input that are no longer needed, and the output gate decides when to extract a specific input from the long-term state.

The current input and the previous output, also called short-term state, are fed to four different fully connected layers. The ones controlled by a sigmoid function are the layers that control the gates; their outputs range between 0 and 1 and are fed to element-wise multiplication operations; in this way, if they output is zero, they close the gate, while if the output is one, they open it. The forget gate controls which parts of the long-term state should be erased, the input gate controls which new memories should be added to the long-term state, and the output gate controls which parts of the long-term state should be read and output at this time step. The new memories are calculated in the layer controlled by the *tanh* function.

### Neural Network Implementation

In order to evaluate the best neural network architecture, two different setups were implemented:

- *Direct SBP/DBP prediction*: The network analyses 5 s of recording and then directly outputs a single value for SBP (peak) and another one for DBP (valley);
- *Entire ABP signal prediction*: the network predicts the continuous blood pressure signal in real-time.

Predicting the entire signal would be better for clinical application, while for commercial healthcare device implementation, only systolic and diastolic values are predicted.

Every neural network configuration was trained in the two setups with both datasets and evaluated on a validation set; then, the best performing networks were cross-validated using the method of Leave-One-Out (LOO) since it is the most robust approach in terms of generalization performance [1]. ANNs were trained by using the Adam optimizer, learning rate η = 0.001, Huber loss, and mini-batch training; they were implemented in Tensorflow 1.15, and the training graphs were visualized through Tensorboard (the official Tensorflow visualization tool). Since Adam is an adaptive learning rate algorithm, it did not require a lot of tuning; therefore, the default learning rate was used. The Huber loss was chosen because it is a robust metric, unaffected by outliers, considering that the dataset has no bell-shaped distribution. Finally, samples of recorded PPG have different dimensions between the two setups: samples in *direct SBP/DBP prediction* are 5 s long, while in *entire BP prediction* are 2 s long. This difference is due to LSTM, which has problems to manage too long sequences, even though it performs better than classic RNN. LSTMs are used also in the first setup; however, in this case, it was possible to downsample the input through convolutional layers, since it was not necessary to output a value for every input.

In *direct SBP/DBP prediction*, recordings were divided into 5 s chunks; then, the algorithm developed in Elgendi et al. [32] was employed to extract SBP and DBP values. Since in 5 s there are usually 4 to 6 cardiac cycles, the mean SBP and mean DBP were taken as the target values.

## Direct SBP/DBP Prediction

### ResNet

**Fig. 17:**
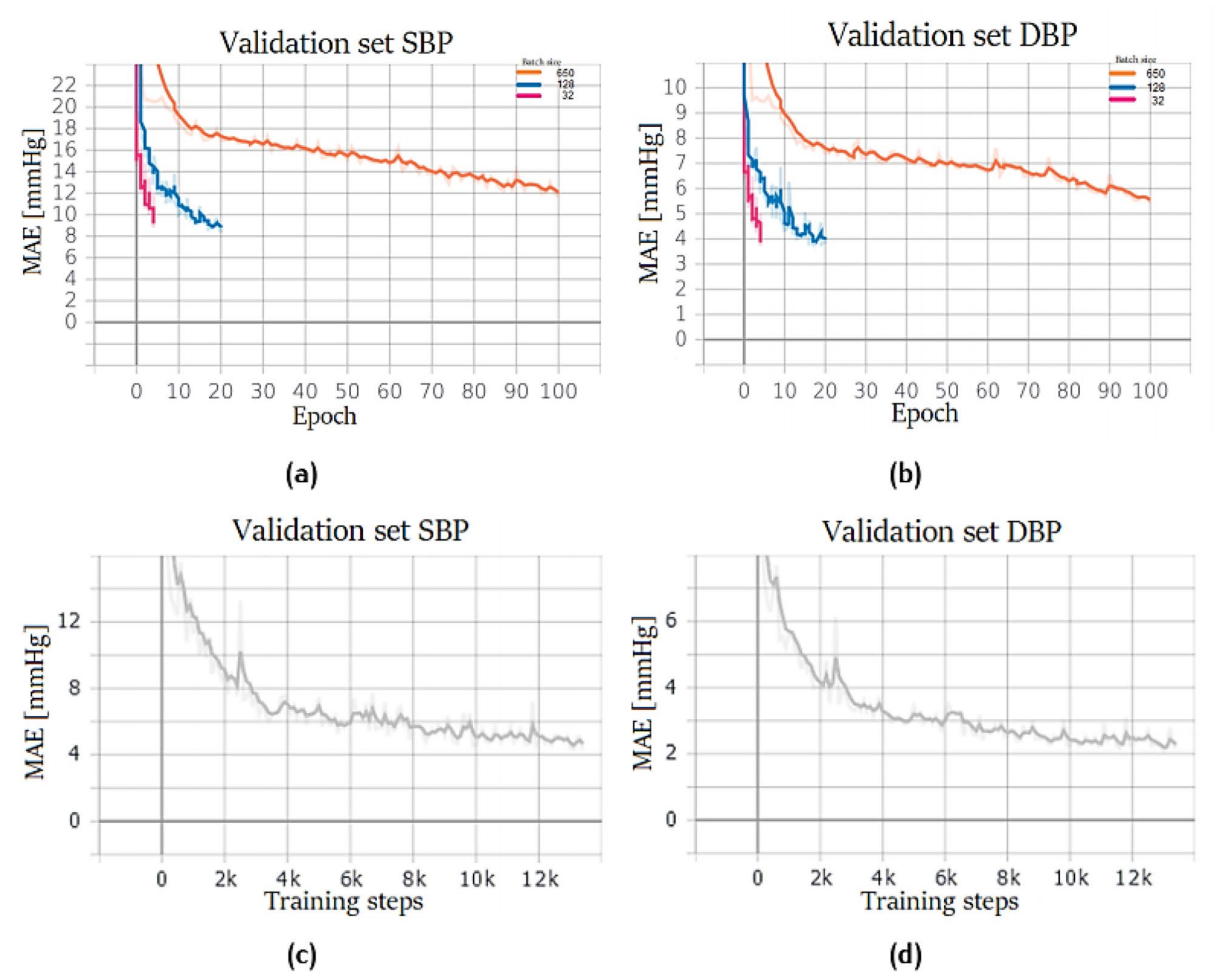
ResNet training performance. Network trained on PPG dataset for SBP (**a**) and DBP (**b**) prediction with different batch sizes: 650 (orange), 128 (blue), and 32 (red). Network trained on PPG + ECG dataset for SBP (**c**) and DBP (**d**); batch size equal to 32

The first attempt, shown in Fig. 17a–b, was made by using a ResNet18 and testing different batch sizes. Smaller batches allowed a faster training and achieved better results, probably because they did not get stuck in some local minimum. Therefore, also for a regression task, mini-batch training is the best way to train a neural network. In particular, three settings were tested: in the first, 650 samples per batch were used (i.e. maximum size permitted by Google Colab GPU), before performing backpropagation; in the second, 128 samples, and in the third 32 samples were adopted. In every setup, the number of training steps is always the same: this is important because it represents the number of times the weights are updated; therefore, the networks are comparable only if their weights are updated the same number of times:

7 $$Training steps=epochs* \frac{Number of samples}{Batch size}$$

Classical feature selection has been automated by convolutional layers and skip connections; in addition, it is possible to stack layers creating a deep neural network, which can better analyse input data.

Once the best batch size was chosen (32 samples), a network was trained also on the dataset composed of PPG and ECG (see Fig. 17c–d) signals.

### ResNet and LSTM

The next experiment employed a ResNet, like the previous one, followed by three LSTM layers, each one made up of 128 neurons. The first LSTM layer is bidirectional. Convolutional layers are activated when they are combined with recurrent layers: they extract features from a signal, and they can also downsample the input sequence using the right kernel size, stride, and padding. The model can learn how to preserve the useful information dropping only the unimportant details and shortening the sequences; the convolutional layer may help the following recurrent layers to detect longer patterns. This network was the best performing to directly predict SBP/DBP values for both datasets.

**Fig. 18:**
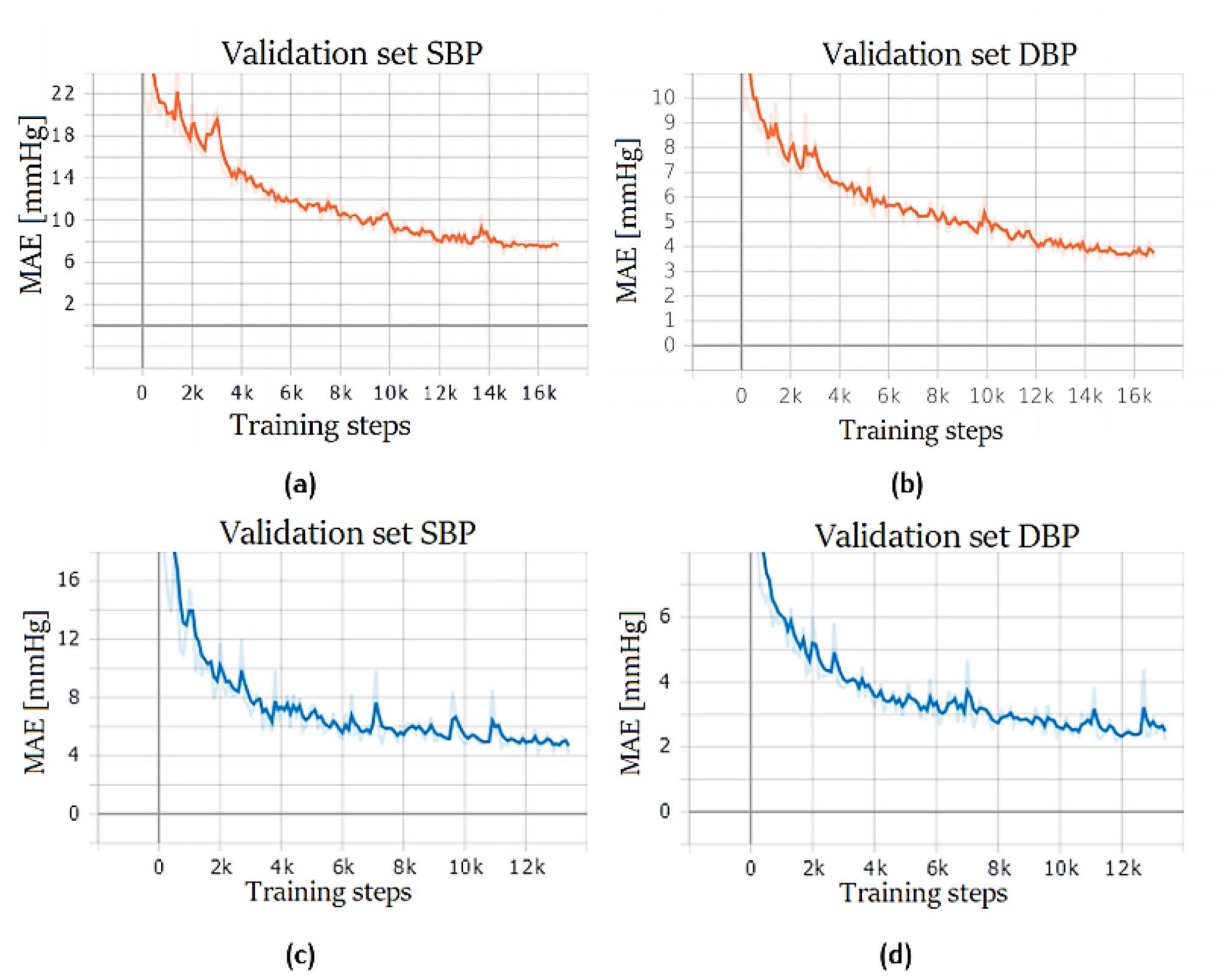
ResNet + LSTM training performance. Network trained on PPG dataset for SBP (**a**) and DBP (**b**) prediction. Network trained on PPG + ECG dataset for SBP (**c**) and DBP (**d**)

Training results are shown in Fig. 18: since the two datasets have different number of samples, the networks were trained with a different number of training steps.

## Entire ABP Signal Prediction

### Fully Connected Network

**Fig. 19:**
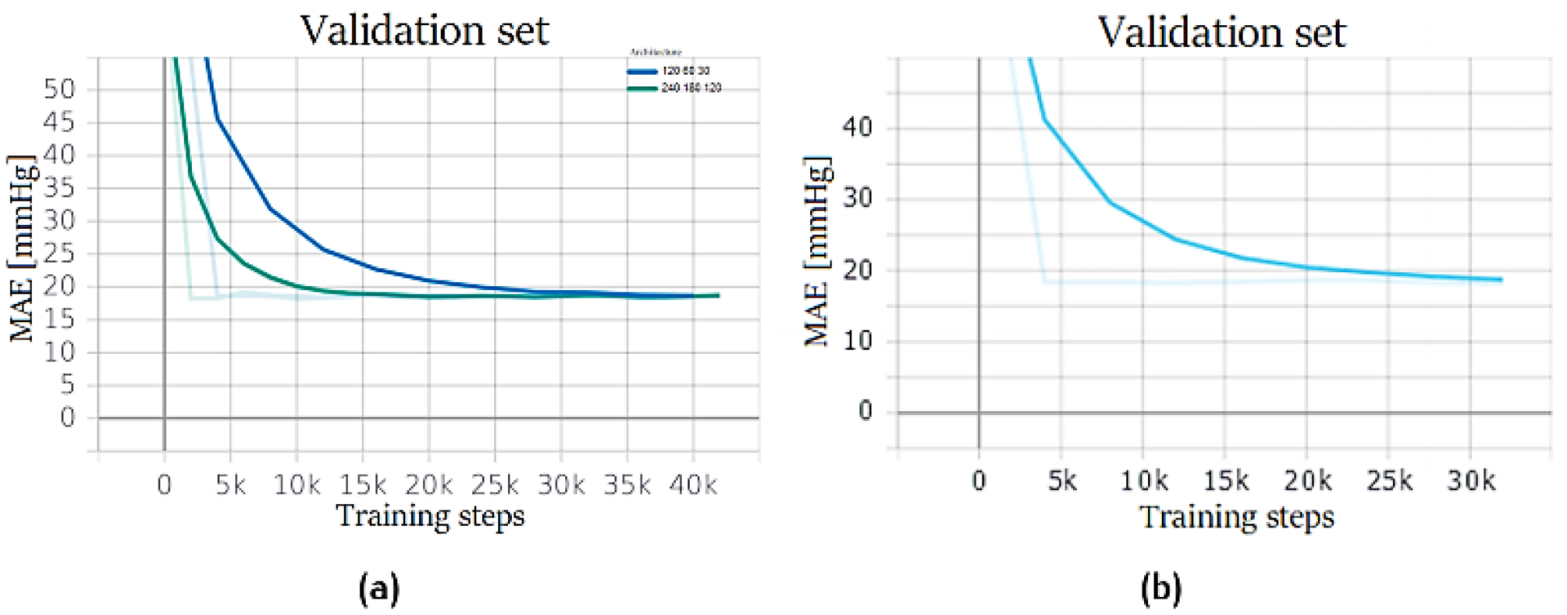
Fully connected training performance. Network trained on PPG dataset with different number of neurons (**a**): 120–60-30 (blue), 240–180-120 (green); network trained on PPG + ECG dataset (240–180-120) (**b**)

It was tried to predict ABP signal through a simple fully connected neural network. Different architectures were implemented; however, due to the simplicity of the model, good results were not achieved. Deeper models appear to converge faster, but still give high errors, as shown in Fig. 19a. PPG + ECG datasets were used for training only on the deepest model; however, results did not improve significantly (see Fig. 19b).

### Long Short-Term Memory

**Fig. 20:**
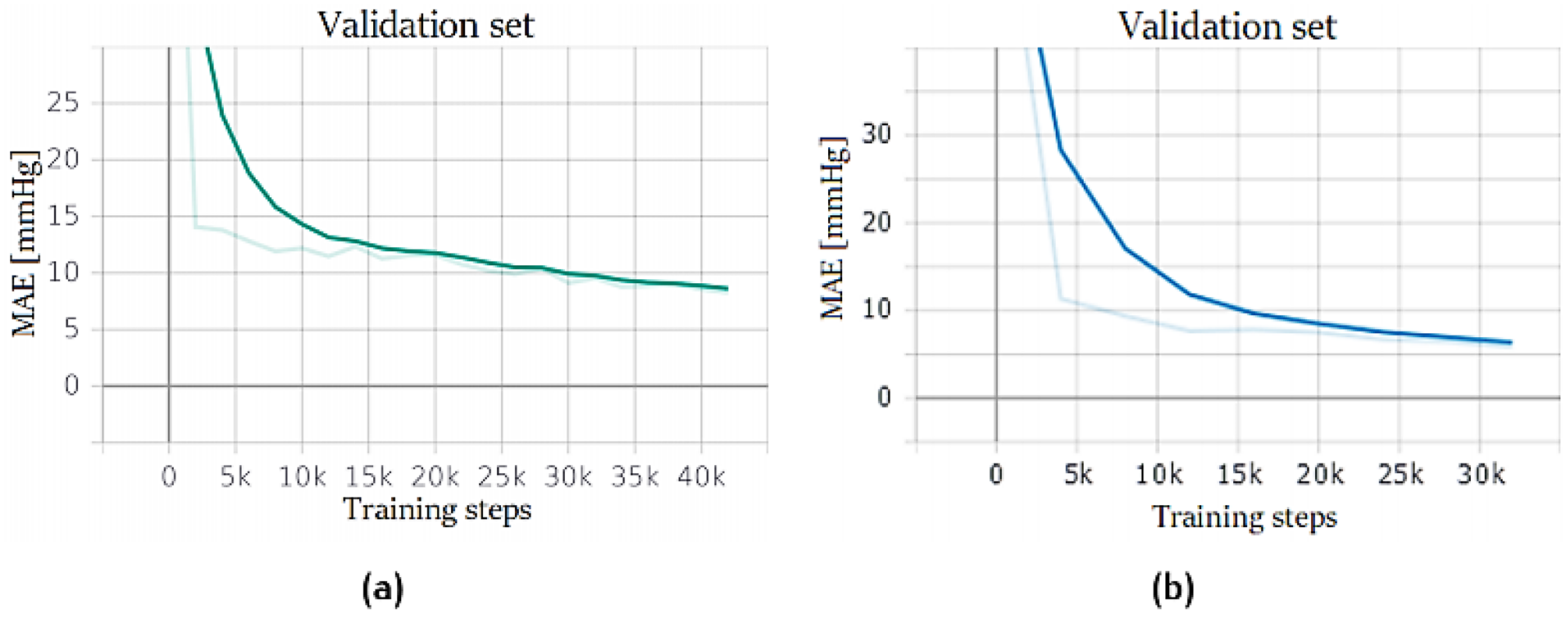
LSTM stack training performance: network trained on PPG dataset (**a**) and PPG + ECG (**b**)

The network is composed of three stacked LSTM layers, each one with 128 cells. The first layer is bidirectional, while the output layer is a fully connected neuron without any activation function. Bidirectional Long Short-Term Memory (BLSTM) looks for contextual features both forward and backward, which is useful because the location of the feature that the network wants to forget is not known. This approach is used also by humans every day: sounds, words, and even whole sentences that at first mean nothing are found to make sense in the light of the future context; in practice, they are used to increase the amount of input information available to the network. This is the approach widely used in natural language processing, and it was successfully used also in ABP prediction by several researchers [27]. BLSTM usually is placed as the first layer of the network because it has access to a much larger-scale context of the input sequence. BLSTMs heavily increase the computational cost; thus, it is reasonable to use only one bidirectional layer. Every sample is composed of 2 s of recording; as explained earlier: this of the value length was defined because LSTMs have problems to manage long sequences. It is hard to remember long-term patterns if the sequence is too long; furthermore, long sequences imply a deep unrolled network, which makes too hard the computation of the gradient through time. For this reason, only the 2 s before the current instant *t* are taken as an input time window to predict a single output value at the instant *t*. Training results are shown in Fig. 20.

### WaveNet

**Fig. 21:**
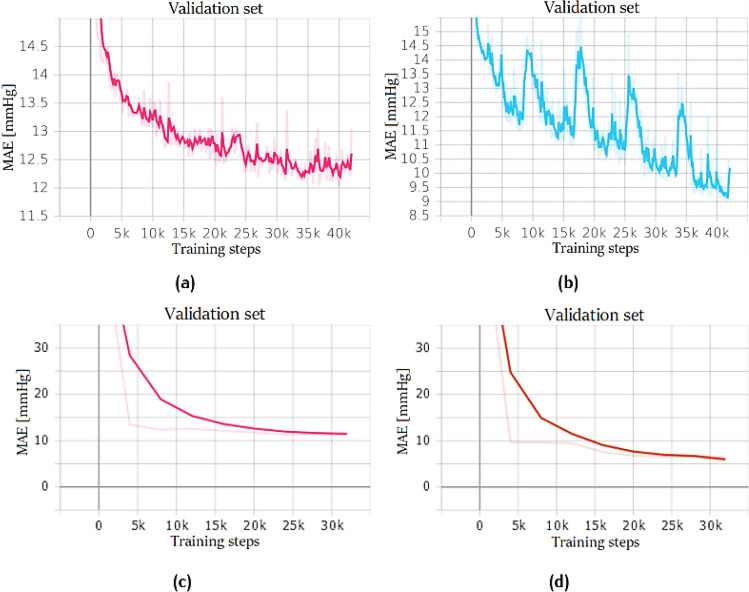
WaveNet and WaveNet + LSTM training performance. PPG Dataset: WaveNet (**a**) and WaveNet + LSTM (**b**). PPG + ECG dataset: WaveNet (**c**) and WaveNet + LSTM (**d**)

Another experiment used a simplified version of the WaveNet, composed of two blocks each one with four convolutional layers. Dilation rate is the double (from 1 to 8) in every convolutional layer inside a block. The output layer is a fully connected neuron without any activation function. Since the network is composed only by convolutional layers, it converges fast and, thanks to the doubling dilation rate, it can process extremely large sequences very efficiently. Afterwards, a second network was built stacking three LSTM layers, each composed by 128 neurons, where the first layer was bidirectional. On top of this simplified WaveNet, convolutional layers extract features that are then analysed by LSTM layers. Training results are shown in Fig. 21.

### ResNet and LSTM

**Fig. 22:**
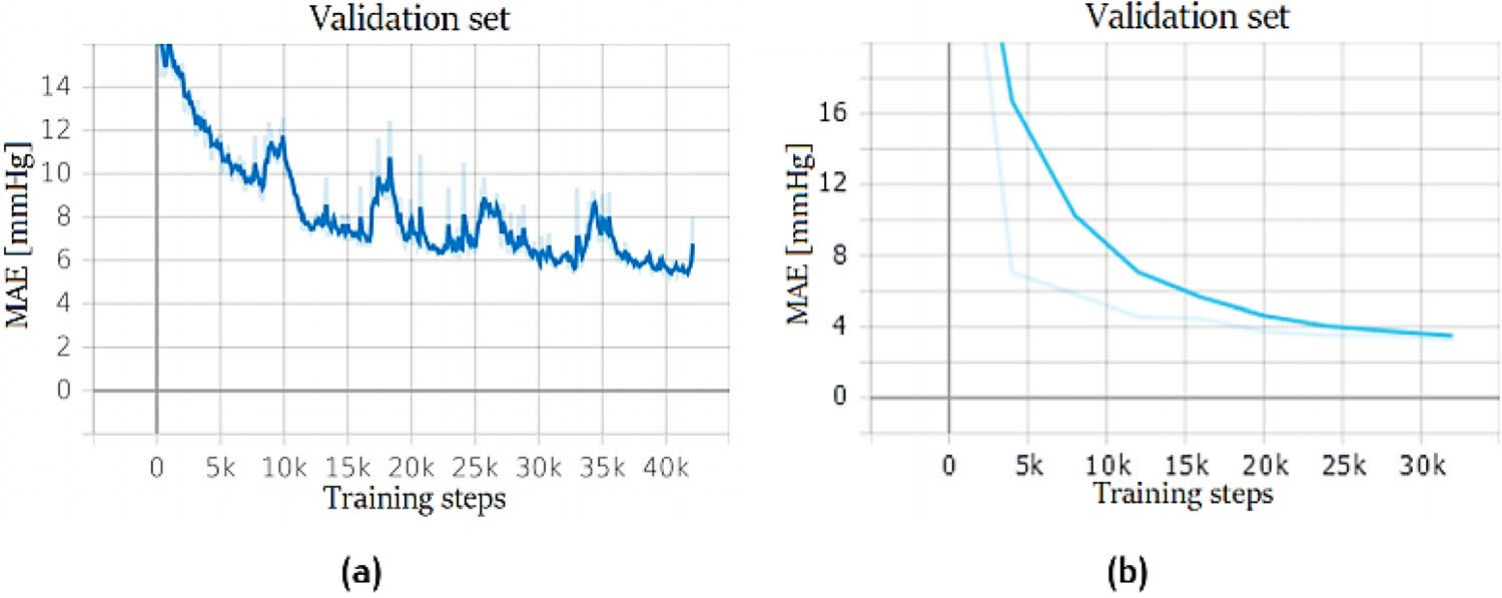
ResNet + LSTM training performance: PPG dataset (**a**), PPG + ECG dataset (**b**)

**Fig. 23:**
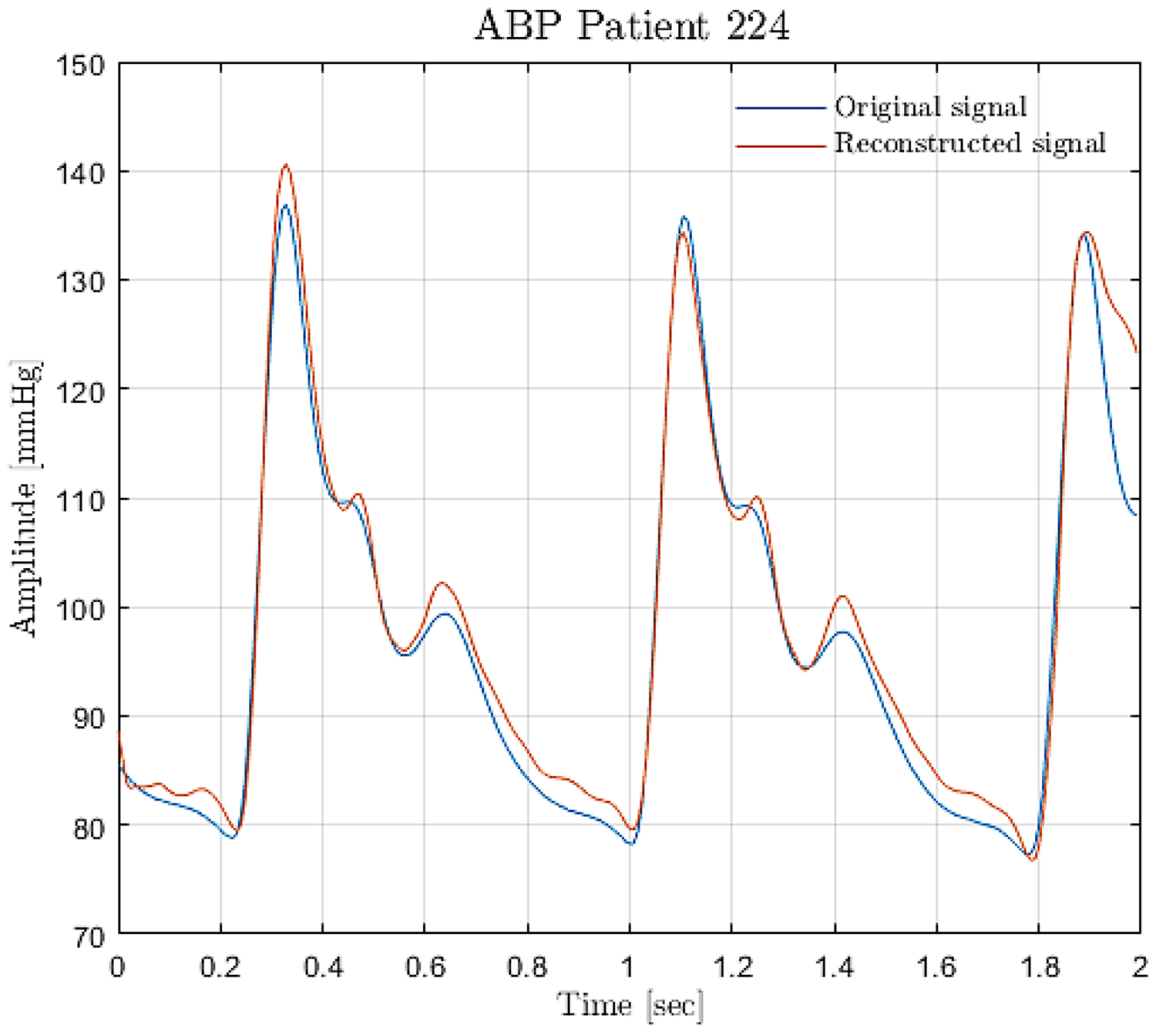
BP Prediction on a validation set sample made with ResNet + LSTM trained with PPG dataset: original signal (light blue) vs network output (orange)

Finally, since using LSTMs layers on top of convolutional layers was proven to be a good approach, an attempt was made to use a deeper model: a modified ResNet followed by three LSTM layers, the first one bidirectional, whose results are shown in Figs. 22 and 23. This network is different from the one presented in section *direct SBP/DBP prediction* because max-pooling layers are not used and convolutional layers have causal padding, like WaveNet. This is a crucial step: in order to predict the entire signal, it was necessary to output a sequence of the same length as the input sequence. This network achieved the best performance in predicting the entire signal. In this paper, every ResNet is composed by four ResNet blocks. Convolutional layers have kernel sizes equal to 3 and strides equal to 2, while the number of filters increases in every block starting from 64 up to 512. In particular, this ResNet is followed by three LSTM layers, the first one bidirectional. Every layer is composed of 128 cells. Although by default, Keras uses Glorot initialization with a uniform distribution to reduce the risk of exploding/vanishing gradients at the beginning of training; this is not enough to solve this problem during training. For this reason, every convolutional operation is here followed by batch normalization, which zero-centres and normalizes each input; afterwards, it scales and shifts the results by using two new parameter vectors per layer: one for scaling, the other for shifting. In other words, this procedure makes the model learn the optimal scale and mean of each of the layer inputs [35]. This network was the best performing network to predict the entire BP signal for both datasets. The network was built ad hoc on this problem. Very simple convolutional neural networks and recurrent neural networks were initially trained but, as reported in “Results,” the hybrid approach between CNN and RNN produced the best results. The transfer learning approach has not been explored in this case, but will be applied in future works.

### Leave-One-Out

**Fig. 24:**
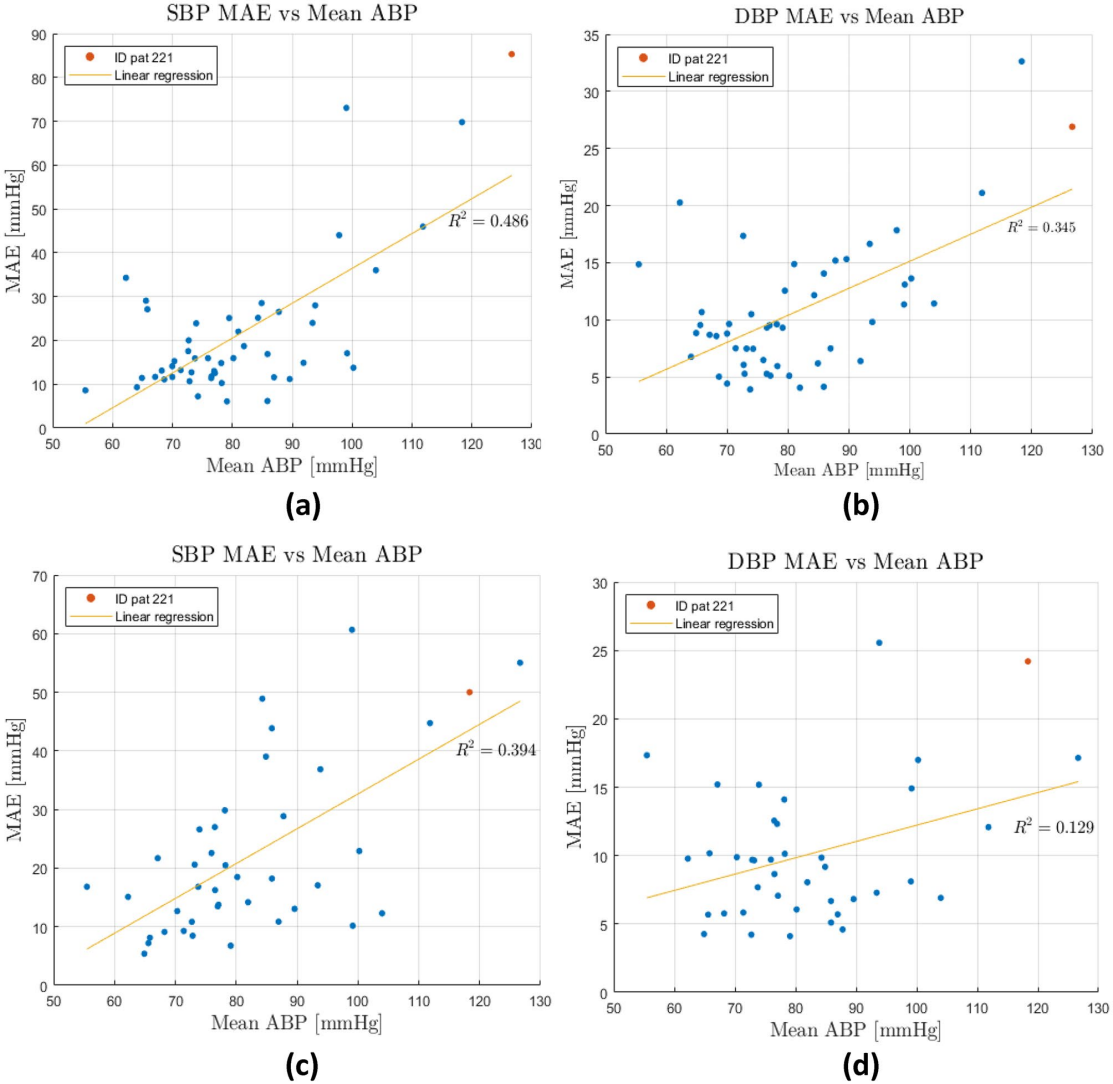
“Entire BP prediction” LOO error for different patients depending mean ABP; **a** and **b** refer to dataset with only PPG and **c** and **d** to dataset with PPG and ECG

**Fig. 25:**
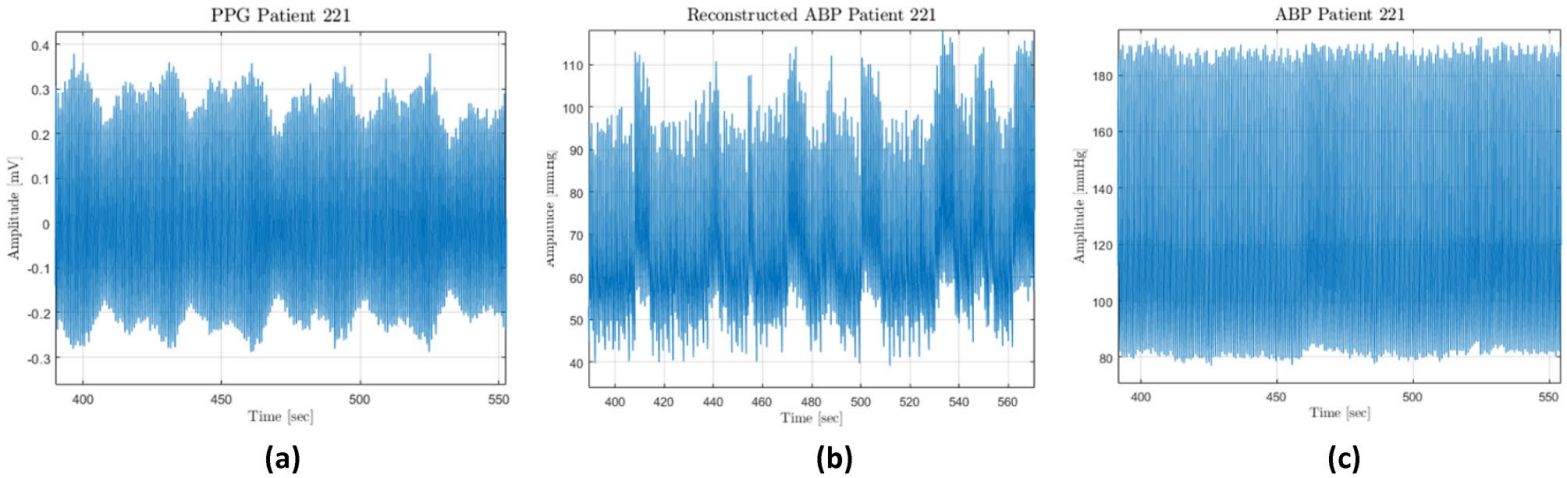
NN tends to predict an ABP with similar pattern to those in PPG, while real ABP does not have them

In order to understand the generalization performances, a Leave-One-Out (LOO) cross-validation was conducted on the best architecture, i.e. ResNet followed by LSTM, for both datasets: the one built using only PPG and the one built using both PPG and ECG. This method was chosen because it tests every possible way to divide the original sample into training and validation, and it has lower computational cost compared to the alternatives [37]. The overall errors were computed as the average of individual MAEs in each LOO iteration. The results were worse than the ones obtained with the same network trained and tested on the same patients, which means that personalization boosts the predictions. There is a correlation between mean ABP and the errors (see Fig. 24). Since the datasets have a majority of physiological ABP, when the network is trained with a great majority of healthy ABP and then it is used to predict an unhealthy ABP, the error is greater than it should be. In addition, there are some long patterns in some PPG (see Fig. 25), which cannot be recognized by the network because they are longer than the training sample length (2 s). By using the ECG signal, the network performance is improved on the validation set and makes the results more general; indeed, it performs better also on LOO cross-validation and the predictions are less dependent on mean ABP.

### Polito Dataset

Finally, a custom dataset was created at Neuronica Lab of Politecnico di Torino to test the proposed algorithm. Nine healthy subjects (5 males, 4 females, aged 22.84 ± 1.07 years) were recruited to participate in the experiment of PPG, ECG, and BP signal acquisitions. The recordings were gathered using a GE Healthcare B125 patient monitor, which is a certified clinical device, generally appreciated for its intuitiveness and reliability in a variety of acuities. The monitor delivers proven NIBP technology, utilizing GE-patented “smart cuff” pressure control to improve measurement time, patient comfort, and artefact rejection. It meets the requirements expected by both AAMI ISO81060-2 and IEC 80,601–2-30. Time of day and ambient temperature were not strictly controlled, although most recordings were made in the morning. Volunteers were seated and put at ease so that the commitments of everyday life would not affect the recordings. PPG, ECG, and ABP were measured three times by using the following recording protocol: first, the PPG and ECG were recorded simultaneously; then, ABP was measured using a sphygmomanometer. The PPG and ECG recordings were 15 s long. PPG was sampled at 300 Hz, while ECG at 100 Hz; thus, both signals were resampled both at 125 Hz, with downsampling (samples were skipped) and linear interpolation method, respectively. A sphygmomanometer was used because a CNAP system was not available, while invasive methods can only be performed by trained personnel. Only ECG lead I was recorded, because the developed algorithm is designed to be embedded in a wearable device, which typically only measures this lead.

In this scenario, an additional dataset was derived from MIMIC using PPG, ECG lead I, and ABP. The previous dataset, created by using lead V, demonstrated how the ECG could improve the performance of a neural network that has to predict ABP without having to deal with a small dataset. Of course, the networks trained with lead V could not be reused on the Polito dataset because their weights were not trained for lead I; thus, it was necessary to create a new dataset to train the network. The new MIMIC dataset consisted of 12 patients to which the same pre-processing scheme was applied as before. Then, this dataset was used for training the previous best performing NN architecture: *direct SBP/DBP prediction ResNet* + *LSTM*.

**Fig. 26:**
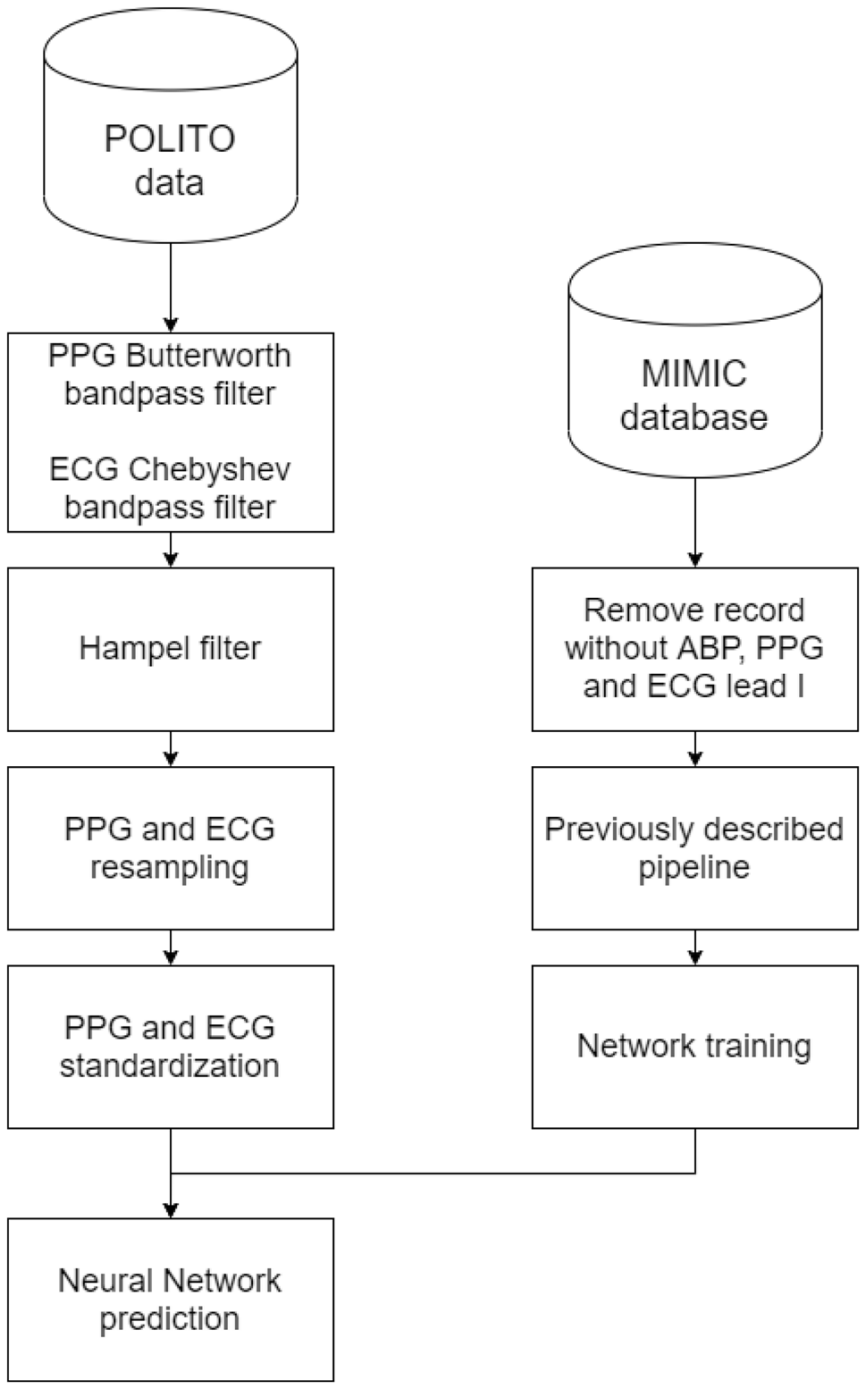
Pipeline used to process never seen data (Polito dataset)

The scheme adopted to predict BP starting from a dataset never met before is shown in Fig. 26.

## Results

Table 1 summarizes the performances of SBP and DBP prediction obtained with the two different setups and networks. Using both PPG and ECG improved the performance in every configuration. In particular, the best network was the ResNet + LSTM, which directly predicted SBP and DBP values. The network overall MAEs on the validation set were 4.118 and 2.228 mmHg. Errors were lower on DBP, because it had lower variability relative to SBP. As expected *direct SBP/DBP prediction* seems to be the best approach if the goal is just to output SBP and DBP values, because the networks are tailored for this purpose. On the contrary, when the networks have to infer the entire signal, they have to learn pieces of information that will not be used. Another advantage of *direct SBP/DBP approach* is the possibility to analyse longer sequences, thus recognizing longer patterns. Applying the algorithm [32] on a predicted ABP signal may introduce further errors. Nevertheless, *entire BP prediction* is an interesting approach for its clinical application and its results are fully shown in Table 2. Finally, LOO cross-validation was employed on the best performing networks for both setups. From Table 1, it is clear that the best network is the ResNet + LSTM in both cases.

**Table 1:** Errors (mmHg) on SBP and DBP prediction for different setups with MIMIC database

Neural network (training dataset)	SBP	DBP	SBP	DBP
	MAE		RMSE	
Direct SBP/DBP prediction				
ResNet (PPG)	9.556	4.217	13.572	6.012
ResNet (PPG + ECG)	4.667	2.445	6.227	3.042
ResNet + LSTM (PPG)	7.122	3.534	11.214	5.029
ResNet + LSTM (PPG + ECG)	4.118	2.228	5.682	2.986
Entire BP prediction				
Fully connected (PPG)	36.559	10.602	45.013	13.417
Fully connected (PPG + ECG)	29.753	12.759	39.330	15.198
LSTM (PPG)	12.118	5.018	17.875	6.890
LSTM (PPG + ECG)	7.603	3.688	11.846	5.320
WaveNet (PPG)	18.539	8.154	26.638	11.441
WaveNet(PPG + ECG)	14.501	7.224	22.922	10.477
WaveNet + LSTM (PPG)	14.353	6.311	21.323	9.150
WaveNet + LSTM (PPG + ECG)	8.812	3.471	12.967	4.864
ResNet + LSTM (PPG)	8.660	3.843	13.439	5.718
ResNet + LSTM (PPG + ECG)	4.507	2.209	6.414	3.101

**Table 2:** Errors (mmHg) on entire BP prediction for different setups with MIMIC database

Tested set	MAE	RMSE	MAE	RMSE
	PPG		PPG + ECG	
Fully connected	18.547	27.214	18.329	25.740
LSTM	8.591	13.306	5.897	9.321
WaveNet	12.292	18.297	11.338	17.518
WaveNet + LSTM	10.009	15.610	5.658	8.919
ResNet + LSTM	6.230	8.883	3.282	5.010

As a summary, Table 1 collects the results obtained with the two approaches: in the former, the predicted output value is represented by two discrete values, systolic and diastolic blood pressures; in the latter case, the goal is to obtain the entire pressure wave; thus, after the prediction of the signal, the peaks (systole) and the valleys (diastole) are extracted. Subsequently, the point values of systolic and diastolic pressures are compared. However, as the peak and valley extraction algorithm could introduce error, the error was calculated on the entire output pressure signal (compared with the target signal), as reported in Table 2.

LOO was performed twice on the PPG trained network because, as explained in “Leave-One-Out,” there are two different datasets; in particular, the dataset created using only PPG had 50 patients, while the dataset created using PPG and ECG had only 40 patients. During the training phase, it was important to have access to as much data as possible; thus, every data available was used; conversely, to compare performance, it was useful to have the same dataset. The ECG signal improved also generalization when employed in the best model ResNet + LSTM, as shown in Table 3; however, errors were higher than when the networks were trained and tested on the same patients (different recordings). This phenomenon appears in several other types of research [1, 23], and it is generally called personalization. With individual calibration, PPG and ECG can be used to directly estimate SBP and DBP on new data obtained from the same individual. According to the American National Standards Institute (ANSI) for the “Development of Medical Instrumentation”[33], in order to validate a new device, there should be an average difference of 5 ± 8 mmHg between the standard and the new developed device [27]. The root-mean-squared error for SBP is 5.682, while for DBP is 2.986.

**Table 3:** LOO results on MIMIC Database with the best neural network (ResNet + LSTM), since direct SBP/DBP prediction did not predict the entire signal, the first two columns are empty. Errors are expressed in mmHg. PPG refers to the configuration where only the PPG signal is used as input, while ECG refers to the configuration with both PPG and ECG as input signals

Tested set	MAE	RMSE	MAE S	MAE D	RMSE S	RMSE D
	Direct SBP/DBP prediction					
PPG (50 pat)			23.5976	10.7459	27.6430	12.3444
PPG (40 pat)			24.2227	11.1056	28.2470	12.6419
ECG (40 pat)			20.3667	9.5484	23.0699	10.8475
	Entire BP prediction					
PPG (50 pat)	15.3419	19.1549	21.4666	10.6841	25.3825	12.3489
PPG (40 pat)	15.6788	19.5598	22.4095	10.8180	26.2460	12.4111
ECG (40 pat)	14.6093	18.0184	22.0995	10.1053	24.5865	11.5292

### Polito Database Results

The best performing ANN (ResNet + LSTM) trained on PPG and ECG lead I was used to predict SBP and DBP on Polito volunteers: MAE was equal to 12.435 mmHg on SBP and 8.567 mmHg on DBP (see Table 4, which shows the results for the Polito volunteers dataset collection). The network was trained, also, by using only the PPG. This configuration achieved MAE equal to 9.916 mmHg on SBP and 5.905 on DBP. In this case, ECG improved the performance on data extracted from the MIMIC database but did not affect generalization. Furthermore, it negatively influenced the results on the Polito. The reason is probably due to the small training set; indeed, only 12 patients had ECG lead I in MIMIC database. The unexpected results on the Polito dataset may be due to different pressure acquisition methods. In this case, for technical reasons, the pressure was not acquired with an invasive method, but measured with a sphygmomanometer, which can introduce an epistemic uncertainty. Furthermore, this instrument has an uncertainty of 5 mmHg which has, therefore, introduced additional noise to the measurements, the so-called “aleatoric uncertainty.”

**Table 4:** SBP and DBP prediction errors (mmHg) on Polito database using the best neural network (ResNet + LSTM) trained on MIMIC dataset (built using PPG and ECG lead I)

Tested set	MAE SBP	MAE DBP	RMSE SBP	RMSE DBP
	PPG			
Validation set	7.409	3.706	9.875	4.883
Leave-One-Out	15.706	7.251	17.792	8.171
Polito dataset	9.916	5.905	11.879	7.273
	PPG + ECG			
Validation set	4.546	2.515	5.766	2.982
Leave-One-Out	16.128	6.743	17.875	7.902
Polito dataset	12.435	8.567	14.082	10.211

Table 5 shows the comparison of all the neural networks employed for arterial blood pressure detection in term of complexity. In particular, for convolutional neural networks, the complexity affects the length of the signal, the dimension of the input vector (1 for only PPG input and 2 for PPG and ECG as inputs), and the kernel size, while for recurrent neural network, the complexity affects only the length and the dimension of input vector. The ResNet + LSTM represents the best model in terms of performance, but, at the same time, the most expensive model in terms of computational complexity.

**Table 5:** Neural network complexity comparison

Neural network	Complexity order	Cost estimation (FLOPs)	
		PPG	PPG + ECG
Fully connected	$O(length\times {(vector dimension)}^{2} )$	$\sim 625$	$\sim 2500$
LSTM	$O(length\times {(vector dimension)}^{2} )$	$\sim 625$	$\sim 2500$
WaveNet	$O(length\times {(vector dimension)}^{2} \times kernel size)$	$\sim 1850$	$\sim 7500$
WaveNet + LSTM	$O(length\times {(vector dimension)}^{2} \times kernel size)$	$\sim 1850$	$\sim 7500$
ResNet + LSTM	$O(length\times {(vector dimension)}^{2} \times kernel size)$	$\sim 4375$	$\sim \mathrm{17,500}$

## Discussion

It is possible to perform accurate ABP measurements relying only on PPG; however, the results are influenced by the inter-person variability; to get around this problem and obtain a greater generalization, ECG signals should be considered.

Different people show different ABP and PPG waves; however, the results also depend on the average pressure of the patients: the biggest mistakes were made on patients with the highest mean ABP.

Relationship with mean ABP could be due to the dataset, since most patients had physiological pressures and the distribution of the dataset was not Gaussian. To obtain better results, it would be appropriate to use a larger dataset to have a Gaussian distribution of BP. Large datasets are of utmost importance in deep learning and the reason is clearly shown in Polito results: although the ECG importance was proven, it did not improve the performance, because the network was trained on a very small dataset.

From Table 4, it can be deduced that adding the ECG signal improves the generalization error (leave-one-out) only for DBP, but does not improve SBP errors. The reason can be the presence of higher frequencies in the SBP signal w.r.t. DBP, which implies a more difficult regression problem.

Working with ICU patients, even intra-person variability is a problem; actually, within the same person, there may be sudden changes in pressure that can bring it from physiological to pathological values.

Finally, the LSTMs seem to play a crucial role in the BP analysis, because they take into account the time dependencies (the pressure at a given time cannot differ too much from what was a moment before). For this reason, it is natural that the addition of this layer has greatly improved performance. However, the LSTMs should necessarily be combined with some downsampling method because their memory is still too short to analyse the long patterns that can be observed on the PPG and can affect the ABP.

## Conclusion

PPG-based techniques allow continuous and automated ABP measurements; they are also well tolerated by patients and are cheap and portable. These techniques are based on direct detection of blood volume in the arteries under the cuff. In this study, ECG improved PPG performance in every setup proposed and allowed the network to generalize better: it is therefore important to collect ECG data in deep learning approaches. This system represents a non-invasive, easy technique for blood pressure measurements. Experiments were carried out on a subset of patients from the MIMIC database and a dataset of Polito volunteers. Within-subject validation was compliant with ANSI guidelines: the best performing network achieved a MAE of 4.118 mmHg on SBP and 2.228 mmHg on DBP. The selected network was also tested on a different custom dataset, created at Neuronica Labs (Politecnico di Torino), which achieved better performance than in MIMIC LOO cross-validation. This is probably due to the fact that this dataset was smaller and, therefore, had lower variance. Indeed, the Polito volunteers were all young and healthy subjects, while MIMIC is a particularly complicated dataset, because its patients have a huge variety of pathophysiologies that result in sudden blood pressure changes. Furthermore, in MIMIC, ABP, PPG, and ECG were probably collected with different measurement devices.

The proposed neural algorithm can be embedded in wearable portable devices to perform continuous healthcare monitoring of arterial blood pressure in order to prevent onset of irreversible damage, like cardiovascular diseases and hypertension. Implemented in a device, this algorithm may prove a powerful tool for diagnosing the aggressive covid-19 virus at an early stage.

Future work will focus on three areas. The first will deal with an intensive testing phase of the algorithm on larger datasets such as MIMIC II and MIMIC III. The second will test the algorithm on a database characterized by elderly patients and people with cardiovascular pathologies to demonstrate its validity. The third area will address the danger related to the fact that blood pressure can change suddenly and become dangerous. Since the variability of intra-person arterial pressure is particularly problematic, especially in precarious health conditions, work on detecting this danger is the next challenge.
